# Sulfated Oligomers of Tyrosol: Toward a New Class of Bioinspired Nonsaccharidic Anticoagulants

**DOI:** 10.1021/acs.biomac.0c01254

**Published:** 2021-01-12

**Authors:** Maria Laura Alfieri, Lucia Panzella, Bárbara Duarte, Salomé Gonçalves-Monteiro, Franklim Marques, Manuela Morato, Marta Correia-da-Silva, Luisella Verotta, Alessandra Napolitano

**Affiliations:** † Department of Chemical Sciences, University of Naples Federico II, I-80126 Naples, Italy; ‡ UCIBIO/REQUIMTE and Clinical Analysis Unit, Department of Biological Sciences, Faculty of Pharmacy, University of Porto, 4050-313 Porto, Portugal; § LAQV/REQUIMTE and Laboratory of Pharmacology, Department of Drug Sciences, Faculty of Pharmacy, University of Porto, 4050-313 Porto, Portugal; ∥ CIIMAR and Laboratory of Organic and Pharmaceutical Chemistry, Department of Chemical Sciences, Faculty of Pharmacy, University of Porto, 4050-313 Porto, Portugal; ⊥ Department of Environmental Science and Policy, University of Milan, 20133 Milano, Italy

## Abstract

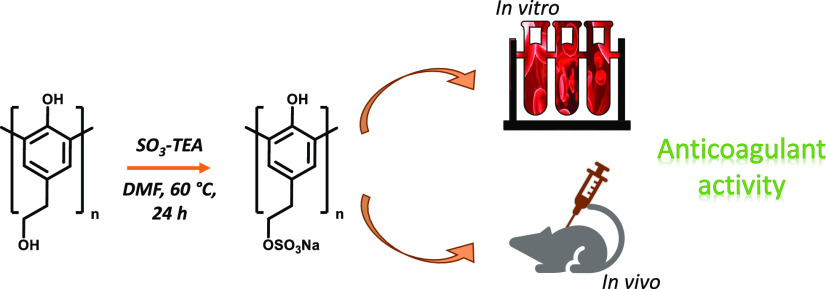

Sulfated phenolic polymers have extensively been investigated as anticoagulant agents in view of their higher bioavailability and resistance to degradation compared to heparins, allowing for increased half-lives. In this frame, we report herein the preparation of sulfated derivatives of tyrosol, one of the most representative phenolic constituents of extra virgin olive oil, by different approaches. Mild sulfation of OligoTyr, a mixture of tyrosol oligomers, that has been reported to possess antioxidant properties and osteogenic activity, afforded OligoTyrS I in good yields. Elemental analysis, NMR, and MALDI-MS investigation provided evidence for an almost complete sulfation at the OH on the phenylethyl chain, leaving the phenolic OH free. Peroxidase/H_2_O_2_ oxidation of tyrosol sulfated at the alcoholic group (TyrS) also provided sulfated tyrosol oligomers (OligoTyrS II) that showed on structural analysis highly varied structural features arising likely from the addition of oxygen, derived from water or hydrogen peroxide, to the intermediate quinone methides and substantial involvement of the phenolic OH group in the oligomerization. In line with these characteristics, OligoTyrS I proved to be more active than OligoTyrS II as antioxidant in the 2,2-diphenyl-1-picrylhydrazyl (DPPH) and ferric reducing/antioxidant power (FRAP) assays and as anticoagulant in the classical clotting times, mainly in prolonging the activated partial thromboplastin time (APTT). After intraperitoneal administration in mice, OligoTyrS I was also able to significantly decrease the weight of an induced thrombus. Data from chromogenic coagulation assays showed that the anticoagulant effect of OligoTyrS I was not dependent on antithrombin or factor Xa and thrombin direct inhibition. These results clearly highlight how some structural facets of even closely related phenol polymers may be critical in dictating the anticoagulant activity, providing the key for the rationale design of active synthetic nonsaccharidic anticoagulant agents alternative to heparin.

## Introduction

Sulfation of phenolic compounds is a common pathway involved in the phase II detoxification of drugs and xenobiotics. Recently, the interest in the design and exploitation of highly sulfated bioactive polyphenols has increased as they have been reported to possess some important biological activities^1^ such as antiplatelet,^2,3^ antiviral,^4−7^ anti-inflammatory,^8,9^ immunomodulatory,^10^ antitumor,^11,12^ and, most importantly, anticoagulant.^3,13−17^ Sulfated polyphenols show effective antithrombotic effects both *in vitro* and *in vivo* (*e.g*., by intraperitoneal administration), high solubility and stability, and low toxic potential.^14,15^

Apart from sulfated small molecules, heparin-mimicking polymers have also been emerging as a valuable alternative strategy for application as anticoagulants.^18−23^ So far, unfractionated heparin (UFH) and its low (LMWH)- and ultralow (ULMWH)-molecular-weight derivatives are the most widespread anticoagulant agents. However, the use of UFH suffers from a number of limitations including enhanced risk for bleeding and thrombocytopenia, unpredictable response, and lack of inhibition of clot-bound thrombin,^24−26^ while the low (LMWH)- and ultralow (ULMWH)-molecular-weight derivatives including fondaparinux, the fully synthetic methyl glycoside derivative of the antithrombin binding domain pentasaccharide sequence of heparin, offer some advantages over UFH, for example, increased half-life and improved bioavailability.^27^

Compared with heparin or sulfated small polyphenols, sulfated nonsaccharidic phenolic polymers could be of value in therapeutics due to their hydrophobic nature that can contribute to improve their bioavailability and could also resist degradation allowing for increased half-lives.^28^ As an example, sulfated polymers obtained by oxidation of hydroxycinnamic acids under biomimetic conditions effectively prolonged activated thromboplastin (APTT) and prothrombin time (PT) with approximately equal potency as low-molecular-weight heparin (LMWH).^16,29,30^

More recently, sulfated low-molecular-weight lignins have been proposed as oligomeric mimetics of LMWH in view of their effective inhibition of the catalytic activity of thrombin and factor Xa, as well as of a series of heparin-binding serine proteases.^31,32^

On the other hand, bioinspired phenolic polymers exhibit a wide range of interesting properties. First of all, they act as antioxidants, but they are also currently exploited for a variety of applications, including preparation of resins,^33−37^ surface functionalization^38^ (*e.g*., for blood-contacting biomaterials and medical device), drug delivery systems,^39^ stabilization of polymers for packaging,^40−43^ and also as additives of biomaterials to favor cell growth and differentiation.^44−47^

Expected advantages with respect to the parent monomers or other small molecules include lower volatility (with reduced adverse effects), greater chemical stability under processing conditions, and lower tendency to be released from the polymer into the contact medium (food, water, *etc*.).

In this context, we recently developed a simple, biomimetic, low-cost procedure to prepare OligoTyr, a mixture of linear oligomers of tyrosol (2-(4-hydroxyphenyl)ethanol, Tyr), one of the most representative phenolic constituent of extra virgin olive oil,^48,49^ using the horseradish peroxidase (HRP)-H_2_O_2_ system.^50^ OligoTyr exhibited osteogenic properties in human osteosarcoma SaOS-2 cells and was found to be significantly more active than tyrosol in several chemical assays.^50^

With the aim to further exploit the potential of OligoTyr, in this work, we prepared sulfated derivatives by different approaches and tested their antioxidant and anticoagulant properties. A mild sulfation procedure was developed with a view to derivatize only the alcoholic OH group of both Tyr and OligoTyr, leaving the phenolic OH group free to keep the antioxidant activity. The sulfated OligoTyr compounds (OligoTyrS I and II) thus obtained proved to be more efficient than the corresponding monomer sulfated tyrosol (TyrS) as antioxidants as well as anticoagulants. OligoTyrS I also showed antithrombotic effect *in vivo* as evaluated by the decrease of the weight of the FeCl_3_-associated thrombus induced in mice treated with OligoTyrS I intraperitoneally in comparison to that developed in the control group. Data from chromogenic coagulation assays showed that the anticoagulant effect of OligoTyrS I was not dependent on antithrombin or factor Xa and thrombin direct inhibition.

## Experimental Section

### Materials

All chemicals were purchased from Sigma-Aldrich and were used without any further purification. OligoTyr was prepared as previously described.^50^ Commercial reagents for the determination of clotting times were purchased from SIEMENS.

### Methods

^1^H NMR and ^13^C NMR spectra were recorded in D_2_O at 400 MHz on a Bruker 400 MHz spectrometer. ^1^H,^1^H COSY, ^1^H,^13^C HSQC, and ^1^H,^13^C HMBC were run at 400 MHz using Bruker standard pulse programs. All samples were exchanged with D_2_O before running the spectra. A given amount (50 mg for carbon spectra and 15 mg for proton spectra) was dissolved in D_2_O (98 atom % D, 0.5 mL), and the solution was taken to dryness under vacuum at around 300 K. The procedure was repeated three times. The sample was eventually dissolved in D_2_O (0.5 mL), and spectrum was run at 298 K. *Tert*-butanol was used as internal standard for ^1^H NMR spectra.

#### Ultraviolet–visible (UV–vis) spectra were recorded on a Jasco V-730 Spectrophotometer

High-performance liquid chromatography (HPLC) analyses were performed on a Shimadzu SCL-10AVP instrument equipped with a UV–vis detector using a Phenomenex Sphereclone ODS(2) C18 column (250 mm × 460 mm, 5 μm), using 1% formic acid–methanol (95:5 v/v) as an eluant, at a flow rate of 0.7 mL/min and detection wavelength of 254 nm.

LC-MS analyses were run on an LC-MS ESI-TOF 1260/6230DA Agilent instrument operating in positive ionization mode (nebulizer pressure, 35 psig; drying gas (nitrogen), 5 L/min at 325 °C; capillary voltage, 3500 V; fragmentor voltage, 175 V; an Eclipse Plus C18 column, 150 mm × 4.6 mm, 5 μm) at a flow rate of 0.4 mL/min using the same eluant as above.

Positive reflectron MALDI-MS spectra were recorded on an AB Sciex TOF/TOF 5800 instrument using 2,5-dihydroxybenzoic acid as the matrix. The spectra represent the sum of 15.000 laser pulses from randomly chosen spots per sample position. Raw data are analyzed using the computer software provided by the manufacturers and are reported as monoisotopic masses.

Elemental analysis was performed at the HEKAtech GmbH-Analysentechnik (Wegberg, Germany).

### Preparation of Sulfated Tyrosol (TyrS)

To a solution of tyrosol (200 mg, 1.44 mmol) in DMF (20 mL), SO_3_-TEA (5 molar equivalents, 7.2 mmol) was added and the mixture was taken under stirring at 60 °C for 24 h. After DMF removal (using a rotary evaporator at 70 °C), the residue was dissolved in 50 mL of water and washed with ethyl acetate (3 × 50 mL). To the combined aqueous layer, 3 M NaOH was repeatedly added till pH 9.0 and the mixture was taken to dryness until the complete removal of TEA (^1^H NMR evidence). Finally, the residue was dissolved in approximately 2 mL of water and purified on a Sephadex G10 column (45 cm × 1 cm) using water as an eluant. Fractions (6 mL each) were collected and analyzed by HPLC, and those containing the product (fractions 6–10) were combined and taken to dryness to give TyrS as sodium salt (167 mg, 48% yield).

ESI^+^/MS: *m*/*z* 263 ([M + Na]^+^)

^1^H NMR (D_2_O) δ (ppm): 2.80 (t, *J* = 6.8 Hz), 4.19 (t, *J* = 6.8 Hz), 6.83 (d, *J* = 8.4 Hz), 7.20 (d, *J* = 8.4 Hz);

^13^C NMR (D_2_O) δ (ppm): 33.9, 69.7, 115.4, 129.8, 130.3, 154.1.

Proton and carbon resonance assignment was based on two-dimensional (2D) NMR analysis (see SI Figures S4–S7).

### Preparation of OligoTyrS I

OligoTyr (150 mg) was reacted with SO_3_-TEA, as described above for TyrS. After removal of TEA, the residue was dissolved in water and desalted by dialysis against water for 4 h using a cellulose membrane (MWCO: 100–500 D; vol/length, 3:1 mL/cm). After freeze drying, the compound was obtained as a yellowish powder. The preparation was repeated four times with a total yield of 98–114 mg at 70 ± 5% w/w.

### Preparation of OligoTyrS II

TyrS (200 mg) was added to 0.1 M phosphate buffer (pH 6.8) (5.5 mL). A peroxidase from horseradish (HRP) solution (163 U/mL, 0.8 mL) and 30% hydrogen peroxide (170 μL) were then added in two aliquots, at 1 h time intervals. After 2 h under vigorous stirring at room temperature, the mixture was desalinized by dialysis against water as above. After freeze-drying, a yellowish powder was obtained. The preparation was repeated three times with a total yield of 115–125 mg at 60 ± 3% w/w.

### 2,2-Diphenyl-1-picrylhydrazyl (DPPH) Assay

The assay was carried out as described.^51^ Briefly, to 2 mL of a 200 μM DPPH solution in methanol, 200 μL of 3 mg/mL water solution of TyrS, OligoTyrS I, OligoTyrS II, or Trolox was added. The mixtures were taken under stirring at room temperature, and after 1 h, the absorbance at 515 nm was determined.

### Ferric Reducing/Antioxidant Power (FRAP) Assay

The assay was carried out as described,^52^ using a 20 mM solution of FeCl_3_ × 6H_2_O in water, a 10 mM solution of 4,6-tris(2-pyridyl)-s-triazine (TPTZ) in 40 mM HCl, and a 0.69 mg/mL aqueous solution of each sample. To a solution made up of 0.3 M acetate buffer (pH 3.6) (3 mL) plus Fe^3+^ solution (300 μL) and TPTZ solution (300 μL), 50, 100, and 150 μL of each sample were added, and after 10 min, the absorbance of all solutions was measured at 593 nm. Trolox was used as the reference compound.

### Anticoagulant Activity in Human Plasma

Human blood was collected from five healthy donors aged between 22 and 28 years without history of bleeding or thrombosis and who had not taken any medication known to interfere with blood coagulation and platelet function for 2 weeks. Nine volumes of blood were decalcified with one volume of 3.8% sodium citrate solution. The blood was centrifuged for 10 min at 2400 g, and the pooled plasma was stored in aliquots at −20 °C until use.

OligoTyrS I and II and the monomer TyrS were dissolved in saline (0.9% NaCl), and the monomer Tyr was dissolved in DMSO (stock solution 14, 400 mg/L). Six concentrations were prepared from stock solution of OligoTyrS I and II: 1200, 800, 480, 192, 76.8, 30.7 mg/L (saline), and only the two highest concentrations were prepared for the monomers TyrS (saline) and Tyr (10% DMSO in saline). Enoxaparin (Lovenox) was diluted with saline to five concentrations: 800, 480, 192, 12, and 2 mg/L. Plasma and solution of the compounds were mixed at a 1:1 ratio. The clotting times were determined in a Sysmex series CA 500—Model CA 540. The procedure used is described below.

#### Activated Partial Thromboplastin Time (APTT)

Citrated normal human plasma (50 μL) was mixed with a sample solution (1:1) and incubated for 1 min at 37 °C. Then, 50 μL of APTT assay reagent (Dade Actin FS Activated PTT Reagent—ACTIN FS; refCode: B4218-20) was added and the mixture was incubated for 3 min at 37 °C. Finally, 50 μL of CaCl_2_ (calcium chloride solution, CaCl_2_; refCode: ORHO37) was added and clotting times were recorded during 600 s.

#### Prothrombin Time (PT)

Citrated normal human plasma (50 μL) mixed with a sample solution (1:1) was incubated for 3 min at 37 °C. Then, 100 μL of PT assay reagent (Thromborel S; refCode: OUHP29) preincubated at 37 °C was added and clotting times were recorded during 600 s.

#### Thrombin Time (TT)

Citrated normal human plasma (50 μL) mixed with a sample solution (1:1) was incubated for 2 min at 37 °C. Then, 100 μL of thrombin solution (Test Thrombin Reagent—TEST THROMBIN; refCode: OWHM13) preincubated at 37 °C was added and clotting times were recorded during 600 s.

### Anticoagulant Activity *In Vivo*

#### Venous Thrombosis Model

The venous thrombosis procedure^53^ was approved by the local (179/2017-ORBEA-ICBAS-UP) and national (003511/2018-DGAV) competent authorities. Male CD1 mice from the i3S Animal Facility (University of Porto, Portugal) aged 8–10 weeks were used. OligoTyrS I was dissolved in saline and given intraperitoneally (8 mg/kg; 100 μL) 15 min before the procedure. Enoxaparin (Lovenox, 4 mg/kg, subcutaneously, 2 h before the procedure) was used as a positive control. Doses and routes of administration were chosen according to previous works.^14,15^ Control mice received no drug through any route of administration, before the procedure. The mice were anesthetized with ketamine (75 mg/kg) + medetomidine (1 mg/kg), the abdomen was opened, the vena cava was isolated just below the left renal vein, and a cotton thread was used to ligate it. Immediately after, a small filter paper (2 mm × 5 mm) steeped in a 35% ferric chloride solution was applied distally to the ligature for 30 min. Then, the formed thrombus was removed carefully and dried at room temperature overnight. The weight of the thrombus was used as a measure of the thrombotic response.

#### Anticoagulant Mechanism

Enoxaparin and OligoTyrS I were administered to CD1 mice in the doses and routes of administration described above. The control mice received no treatment. Then, 2 h after enoxaparin administration and 1 h after OligoTyrS I administration, the mice were anesthetized with ketamine (75 mg/kg) + medetomidine (1 mg/kg) and nearly 1 mL of blood was collected directly from the heart into syringes containing citrate (1:9). The blood was immediately centrifuged twice at 2500*g*, room temperature, for 20 min. Finally, the plasma was collected and kept at −20 °C until use. Coagulation chromogenic assays on specific coagulation pathways were evaluated in an STA-R Max instrument using STA-LIQUID ANTI-Xa, STA-STACHROM AT III, and STA-ECA II kits from Diagnostica Stago. These coagulation assays were also performed in samples of plasma of control mice spiked with OligoTyrS I (1:10) to achieve the same final concentrations used in human plasma clotting assays (6.25, 12.5, 25, 50, and 100 mg/L). In the STA-LIQUID ANTI-Xa assay, the compound under study competes with factor Xa for the cleavage of a chromogenic substrate; this assay is not only affected by UFH/LMWH/Fondaparinux but also by the direct anti-Xa activity of rivaroxaban, apixaban, and edoxaban. To differentiate the direct or indirect inhibition over factor Xa, the assay is controlled with different calibrators: STA-Quality LMWH was used to evaluate the indirect anti-Xa activity, and STA-Rivaroxaban Control was used to evaluate the direct anti-Xa activity. The STACHROM AT III assay evaluates the capacity of the compound under study to enhance the antithrombin (AT III) activity of the sample to inhibit a known excess of thrombin; the residual thrombin then cleaves a chromogenic substrate. This assay is affected by the presence of UFH as well as by direct thrombin inhibitors, which will directly inhibit the known excess of thrombin. In the STA-ECA II assay, the compound under study competes for the cleavage of a chromogenic substrate, with products that result from the breakdown of prothrombin with ecarin. This assay is used to quantify the new orally active direct thrombin inhibitor dabigatran.

### Statistical Analysis

To evaluate the effect of the compound on venous thrombosis, the mean thrombus weight of each group was compared with that of the control group by Student’s *t* test. The effect of enoxaparin and OligoTyrS I on the coagulation assays was compared with the values found in the plasma of the control mice by Student’s *t* test. A *p* value of less than 0.05 was considered statistically significant.

## Results and Discussion

### Synthesis and Structural Characterization of Sulfated Tyrosol Oligomers

**Scheme 1:**
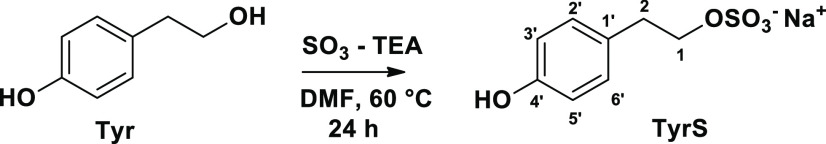
Synthesis of the Monosulfated Tyr Derivative (TyrS)

The conditions of the sulfation reaction were initially optimized on tyrosol, Tyr. A procedure previously reported for the synthesis of sulfated derivatives of hydroxycinnamic acid oligomers,^29,30,54,55^ based on the use of the commercially available sulfur trioxide triethylamine complex (SO_3_-TEA), was initially considered. After several attempts, a procedure was eventually developed involving reaction of Tyr at 70 mM with 5 molar equivalents of SO_3_-TEA in anhydrous dimethylformamide (DMF) at 60 °C for 24 h (Scheme 1). HPLC analysis of the reaction mixture indicated the presence of a main compound (Figure S1), which was obtained from a preparative scale reaction following DMF and TEA removal and gel filtration chromatography in around 50% yield.

NMR (Figures S2–S7) and ESI-MS analyses allowed us to formulate the compound as the sodium salt of the monosulfated derivative of Tyr, TyrS (sodium 2-(4-hydroxyphenyl)ethylsulfate, Scheme 1). The fact that the alcoholic group was the site of sulfation was apparent in the ^1^H NMR spectrum from the low-field shift of the triplet (*J* = 6.8 Hz) due to the protons at C-1 (4.19 ppm) compared to Tyr (3.67 ppm)^56^ and, to a lower extent, of the triplet for the C-2 protons (2.80 *vs* 2.71 ppm for Tyr) (Figure S2). Similarly, in the ^13^C NMR spectrum (Figure S3), the signal for the C-1 carbon was low-field-shifted (69.7 ppm) compared to that of Tyr (63.4 ppm), while the C-2 was high-field-shifted (33.9 ppm) with respect to Tyr (38.2 ppm). On the other hand, the proton and carbon resonances of the aromatic moiety did not show significant changes with respect to Tyr. The lower nucleophilicity of the phenolic group compared to the alcoholic group could in fact account for the lack of sulfation under the reaction conditions adopted.

**Figure 1:**
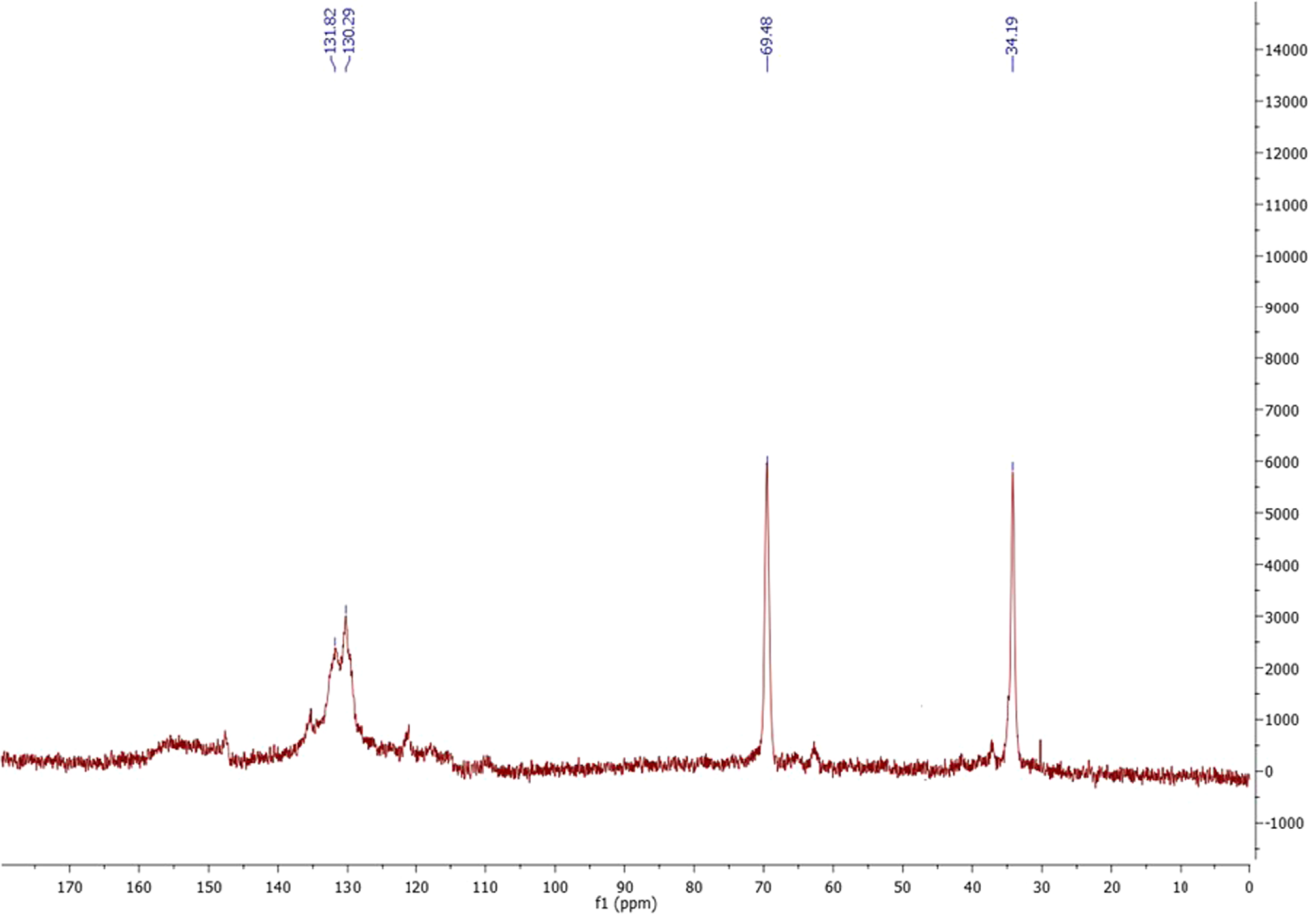
^13^C NMR spectrum of OligoTyrS I in D_2_O.

In further experiments, the developed sulfation protocol was applied to OligoTyr, prepared under the conditions previously described.^50^ The sulfated polymer (OligoTyrS I) was purified by dialysis through cellulose membrane with a 100–500 Da molecular-weight cut-off over a time period (4 h) that had been optimized in terms of minimal loss of material (as monitored by UV–vis analysis) and efficient removal of the saline components (as determined by monitoring changes of specific absorption coefficient of the dried material till constant value). OligoTyrS I was eventually obtained in around 70% w/w yield. Analysis of both the ^1^H and ^13^C NMR spectra showing the diagnostic signals at 4.2/69 and 2.9/34 ppm as the most prominent ones, with the signals attributable to nonsulfated tyrosol units (carbons at 38 and 63 ppm) being very low, suggested a significant extent of sulfation of the hydroxyethyl chains (Figures 1 and S8). As expected for a linear mode of coupling of tyrosol involving the position ortho to the phenolic OH,^50^ the signals at 115 ppm for the C-3′ and C-5′ CH carbons were abated while the resonances at 130 ppm for the C-2′ and C-6′ carbons were prominent in the aromatic region of the carbon spectrum. In agreement with these observations, the S/C ratio determined by elemental analysis for OligoTyrS I (monosodium salt) indicated a high level of monosulfation of the tyrosol units (experimental 0.37 against theoretical 0.33 for OligoTyr sulfated at the hydroxyethyl chain) (Table 1).

**Table 1:** Elemental Analysis for OligoTyrS I and OligoTyrS II (Monosodium Salt)^a^

	% C	% H	% S
OligoTyrS I	31.8 ± 0.22	3.6 ± 0.03	11.7 ± 0.02
OligoTyrS II	19.2 ± 0.15	3.0 ± 0.04	7.52 ± 0.01

a Reported are the mean ± SD of at least three experiments.

The broad signals appearing in the proton spectrum (Figure S8) may likely be attributed to the presence of oligomers of different sizes but with very close structural features or alternatively to heterogeneity of the cations bound. This latter hypothesis was ruled out by the lack of substantial changes of the signal features observed when the spectrum was run after exchange of the sample with ethylenediaminetetraacetic acid (EDTA) (Figure S9). On the other hand, in the carbon spectra, the signals of the aliphatic side chain carbons of sulfated tyrosol units proved indeed much more defined on account of the lower sensitivity of carbon resonances to small differences in the chemical environment, providing support to the hypothesis that the sample consisted of a mixture of regular oligomers.

The sulfated polymer was also subjected to MS analysis in the MALDI-ToF mode using 2,5-dihydroxybenzoic acid as the matrix. After several efforts, a quite defined pattern of oligomer species could eventually be obtained, which confirmed the presence of monosulfated oligomers up to the heptamer (Figure S10). Table 2 shows the tentative structures compatible with the most prominent pseudomolecular ion peaks of the MALDI spectrum.

**Table 2:**
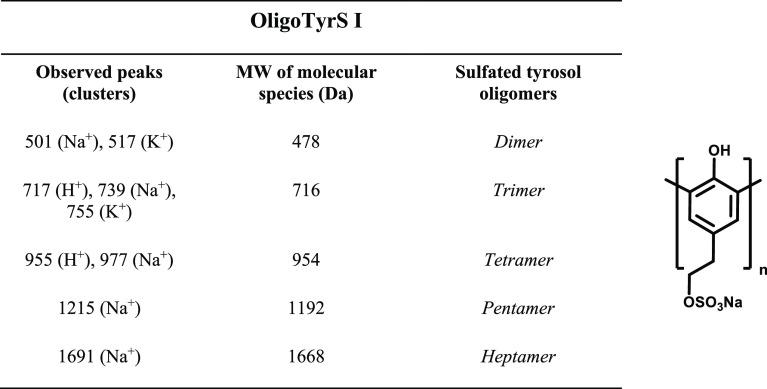
Pseudomolecular Ion Peaks Observed in the MALDI Spectrum of OligoTyrS I and Proposed Structural Assignment

To assess the reproducibility of the sulfation and purification procedure, different batches of OligoTyrS I were prepared. The recovery yields proved comparable (70 ± 5%, *n* = 4) as was the oligomer distribution in the MALDI-MS spectra (Figure S11) and the specific absorption coefficient determined at 280 nm (Figure S12). Furthermore, ^1^H NMR analyses of the batches showed a constant ratio of the integrated areas of the aliphatic/aromatic protons, indicating that the oligomer’s length did not change substantially, nor did the mode of coupling of the tyrosol units, since in the case of high proportions of short-length oligomers, *e.g*., dimers, higher ratios of the areas of aromatic/aliphatic protons would be observed (Figure S13).

In further experiments, an alternative approach was pursued to get a sulfated derivative of OligoTyr based on the oxidation of the monomer TyrS by the HRP-H_2_O_2_ system under the same conditions used for the preparation of OligoTyr. After desalination by dialysis and lyophilization, OligoTyrS II was obtained in around 60% w/w yield (±3%, *n* = 3). The S/C ratio determined by elemental analysis (0.39) (Table 1) indicated a high degree of sulfation, which could be predicted considering that the product is obtained from a sulfated monomer and that the reaction conditions adopted were not likely to allow for removal of the sulfate groups.

However, a relatively higher oxygen content was apparent compared to OligoTyrS I, which could be likely accounted in terms of addition of water and/or hydrogen peroxide to the TyrS units during the oxidation, a process that would be favored by the marked solubility in the reaction medium of both the monomer and the growing oligomers. These secondary reaction pathways would be precluded to OligoTyr, given its immediate precipitation in the reaction medium during its preparation. MALDI-MS analysis failed to provide valuable information except for some signals with low intensity due to sulfated low-molecular-weight oligomers (dimers and trimers) as detected in the spectrum of OligoTyrS I. Some insights into the structural features of OligoTyrS II were obtained by one-dimensional (1D) and 2D NMR analyses (Figures S14–S18).

**Figure 2:**
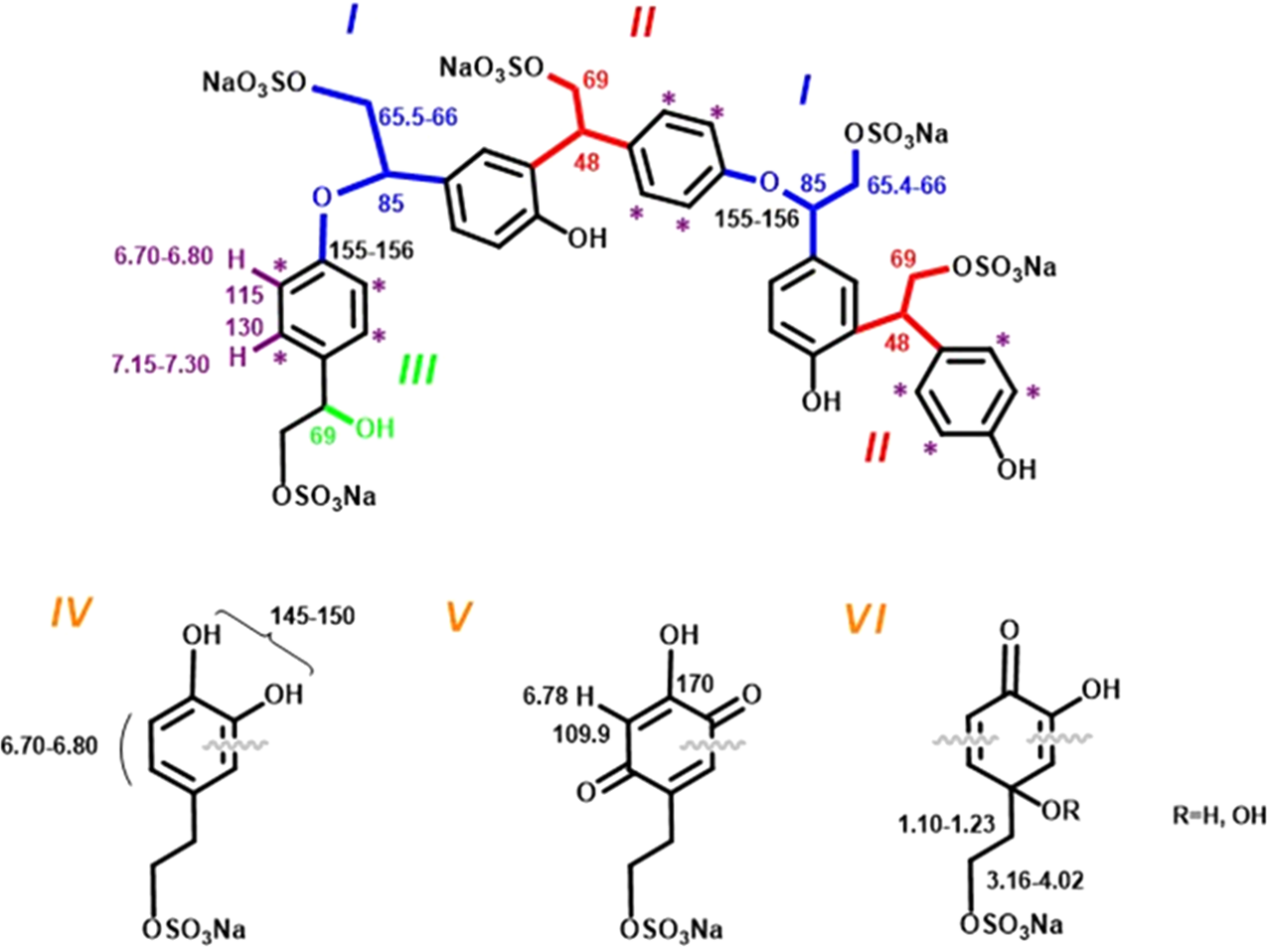
Representative structural components of OligoTyrS II. Highlighted are assignment of resonances based on the analysis of the 1D and 2D NMR spectra.

Notably, the low-field region of the ^1^H NMR spectrum of OligoTyrS II appeared to be more resolved and also more complex than in the case of OligoTyrS I, indicating the presence of structurally related but varied components (Figure S14). In the aromatic region, a series of doublets (coupling constant, 8 Hz) indicated unsubstituted aromatic moieties, *i.e*., not involved in the C–C coupling mode as in the case of OligoTyrS I, whereas the aliphatic region was dominated by two peaks at 2.9 and 4.2 ppm for the sulfated hydroxyethyl chain. The ^1^H,^1^H COSY spectrum (Figure S15) confirmed couplings of the series of aromatic doublets (6.70–6.90/7.11–7.30) and the expected contacts of the sulfated hydroxyethyl chain. Consistent with this conclusion, the ^13^C NMR spectrum showed prominent signals at 115 and 131 ppm, together with several additional signals, both in the sp^3^ and sp^2^ regions, indicative of structural modifications that have occurred in the aromatic rings as well as in the hydroxyethyl chains (Figure S18). These features coupled with consideration of the presence of the aromatic signals as doublets (confirmed also from the ^1^H,^13^C HSQC spectrum showing 7.15–7.30/130 and 6.70–6.80/115 ppm CH correlations) (Figure S16) would suggest that the hydroxyethyl chains could be substantially involved in the coupling between TyrS units. The prominent and multiple carbon signals at around 65 ppm, together with signals at 48 and 85 ppm suggested that branching at the hydroxyethyl chain may have occurred as the result of nucleophilic addition of the reactive position of TyrS, *i.e*., the OH group and the ortho positions, onto the intermediate quinomethides generated in the oxidation as exemplified in partial structural units I and II shown in Figure 2. Another issue that should be considered for interpreting the multiple components of OligoTyrS II is the addition of water or hydrogen peroxide onto the electrophilic position of the quinomethide leading to different structural units of type III or to *o*-diphenols (catechols). The presence of catechol-related units as represented in structures IV–VI is suggested by the observed shielding of some aromatic carbons including the OH-bearing carbons (in the range 145–150 ppm) and also by the presence of shielded protons resonating in the 6.80–6.70 range.

Further to such an event, the addition of oxygen species to the electrophilic sites of the ortho-quinone arising from oxidation of the *o*-diphenol would allow for functionalization of additional positions, *i.e*., position 5′ as in structures *V* featuring a hydroxy-*p*-quinone moiety for which a high-shielded CH carbon at 109.9 bearing a proton at 6.78 ppm and the OH-bearing carbon at 170 ppm could be likely expected, or position 1′ bearing the hydroxyethyl side chain (as in structure VI), the latter allowing also to account for a series of high-field proton signals 1.10–1.23 correlating with a 3.16–4.02 series shown in the ^1^H,^1^H COSY spectrum. Altogether, these reaction pathways would result in a complex mixture of oligomers featuring much diverse coupling modes.

### Antioxidant Properties

**Figure 3:**
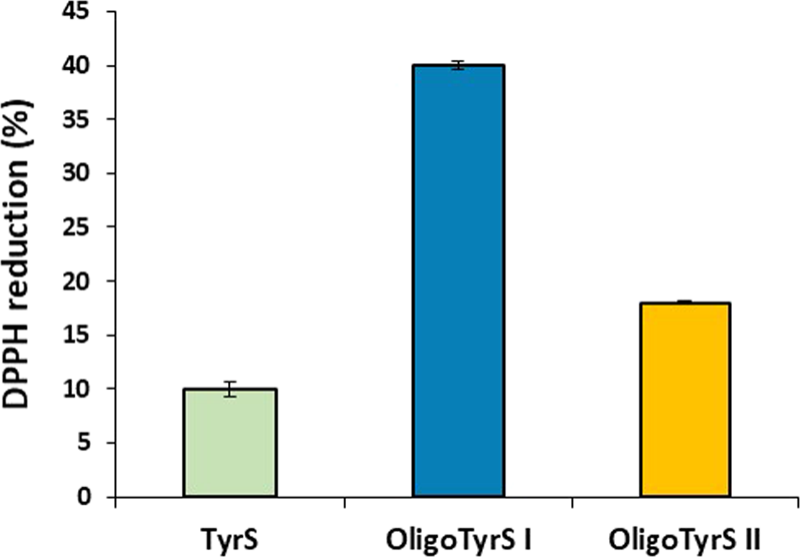
DPPH reduction by TyrS and OligoTyrS species (0.3 mg/mL) after 1 h. Data are shown as mean ± SD of three independent experiments.

**Figure 4:**
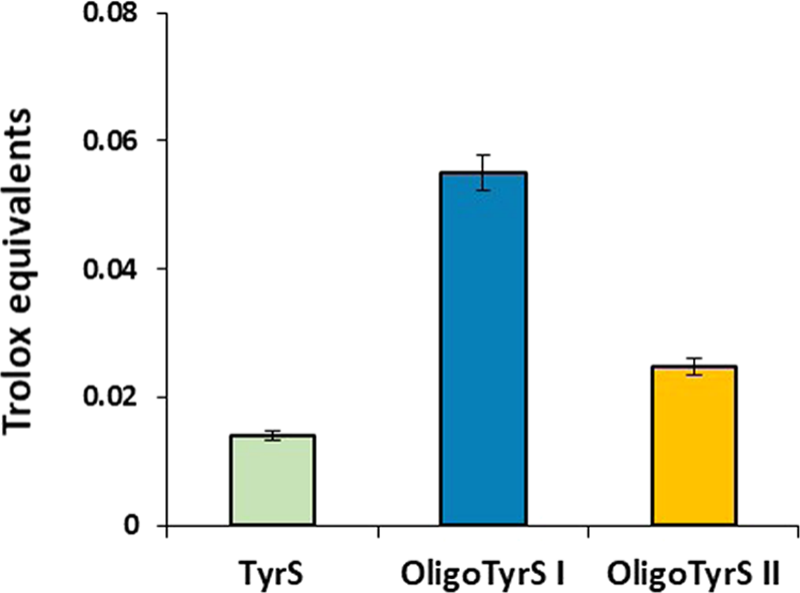
Trolox equivalents determined in the FRAP assay for TyrS and OligoTyrS species. The average values ± SD obtained from at least three separate experiments are reported.

The antioxidant properties of OligoTyrS I and II were evaluated using commonly used assays, that is, the 2,2-diphenyl-1-picrylhydrazyl (DPPH) assay (Figure 3) and the ferric reducing/antioxidant power (FRAP) assay (Figure 4) in comparison to TyrS.^51,52^ Under these conditions, a complete reduction of DPPH was observed using Trolox at the same concentration. In either assay, OligoTyrS I exhibited significantly higher antioxidant properties compared to TyrS, in agreement with that previously reported in the case of OligoTyr, as a result of the stabilizing effect of the aromatic rings at positions ortho to the phenoxyl radical in the oligomeric structures.^50^ Notably, in both assays, OligoTyrS II proved less effective than OligoTyrS I, which would be in line with the high structural heterogeneity of these oligomers and particularly the substantial loss of free phenolic −OH that is likely the major site responsible for the antioxidant effects.

### Anticoagulant Activity

**Figure 5:**
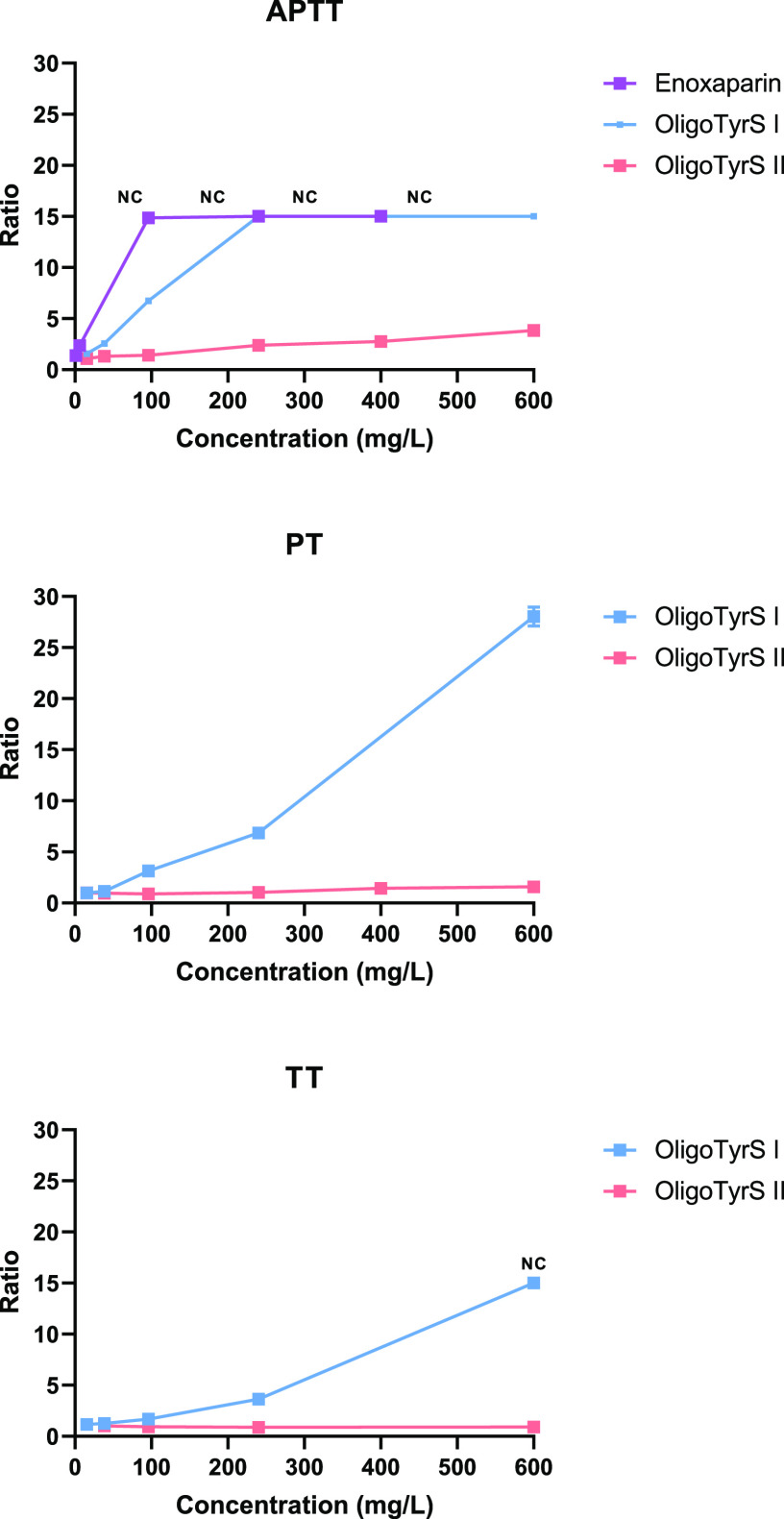
Coagulation time prolonging ratio obtained with OligoTyrS species in the APTT (upper graph), PT (middle graph), and TT (lower graph) tests. Coagulation time prolonging ratio is the ratio of the clotting time when human plasma was spiked with compound solution with respect to that when human plasma was spiked with solvent (control plasma). Enoxaparin (Lovenox) was used as the APTT positive control. NC = no coagulation was observed during the maximum recorded time of the equipment (600 s). In these cases, a value of 600 was arbitrarily assigned and a ratio of 15 for APTT and TT (control plasma = 36 s) and a ratio of 37 for PT (control plasma = 16 s).

The anticoagulant activity was evaluated *in vitro* in human plasma by APTT, PT, and TT clotting assays. The three clotting tests allow us to differentiate between effects on the classical extrinsic or intrinsic pathway or on fibrin formation. Each measurement was performed in duplicate and repeated three times on different days (*n* = 6). Enoxaparin (Lovenox) was used as a positive control. Clotting times obtained in the presence of the monomers TyrS and Tyr (final concentration tested: 600 mg/L) were comparable to clotting times obtained when only their solvents were added to human plasma (data not shown). Differently, OligoTyrS I, OligoTyrS II, and enoxaparin were able to increase the APTT in a concentration-dependent manner, with OligoTyrS I being much more active than OligoTyrS II and less potent than enoxaparin. Indeed, OligoTyrS I was able to total inhibit the APTT pathway from 240 mg/L (no coagulation observed after 600 s), as was enoxaparin from 96 mg/L (Figure 5A). Differently from OligoTyrS II, OligoTyrS I was also able to prolong PT and TT, however to a much lower extent than APTT. The concentration required to double each clotting time (ratio = 2) was calculated from linear regression analysis of each individual concentration–response curve (Table 3).

**Table 3:** Concentration of Tested Compounds Required to Double the Clotting Times Obtained with Control Plasma (ratio = 2)

	APTT_2_ (mg/L)	PT_2_ (mg/L)	TT_2_ (mg/L)
OligoTyrS I	26.19 ± 3.91^a^	50.23 ± 4.96^a^	117.02 ± 1.91^a^
OligoTyrS II	215.91 ± 31.81^a^	n.a.	n.a.
APTT positive control (enoxaparin)	4.07 ± 0.62^a^	n.d.	n.d.

a Standard deviation of three independent experiences performed in duplicate. n.a.= not active at the highest concentration tested; n.d.= not determined.

The concentration of OligoTyrS I needed to double APTT was about 8-fold lower than OligoTyrS II. The higher structural homogeneity of OligoTyrS I compared to the OligoTyrS II could account for the better response observed in the *in vitro* test. Indeed, these structural features have expectedly an impact on the charge density that is higher for OligoTyrS II based on sulfate per unit values determined by elemental analysis, and moreover, the variety of the sulfate-bearing units makes the distance between adjacent charges highly irregular.

The APTT_2_ obtained with the positive control was in accordance with the previously reported values.^57,58^ OligoTyrS I was only 6.4-fold less potent than enoxaparin in APTT, and therefore OligoTyrS I was selected for further *in vivo* studies.

To test the antithrombotic effect of OligoTyrS I, a thrombus was induced in mice that were intraperitoneally treated with OligoTyrS I 15 min before the procedure, and its weight was compared with that formed in the untreated group. Enoxaparin was used as a positive control.

**Figure 6:**
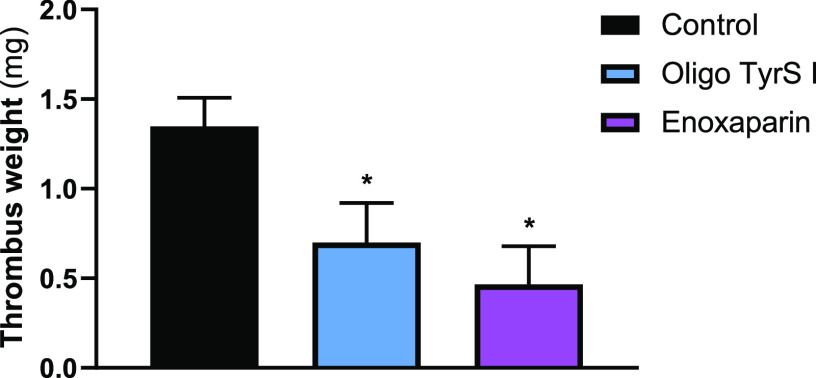
Thrombus weight (mg) formed in each experimental group (*n* = 5 in each). * *p* < 0.05 *vs* control group. Positive control: enoxaparin, subcutaneously.

Both OligoTyrS I and enoxaparin were associated with thrombus of lower weight than those formed in control mice (Figure 6).

**Figure 7:**
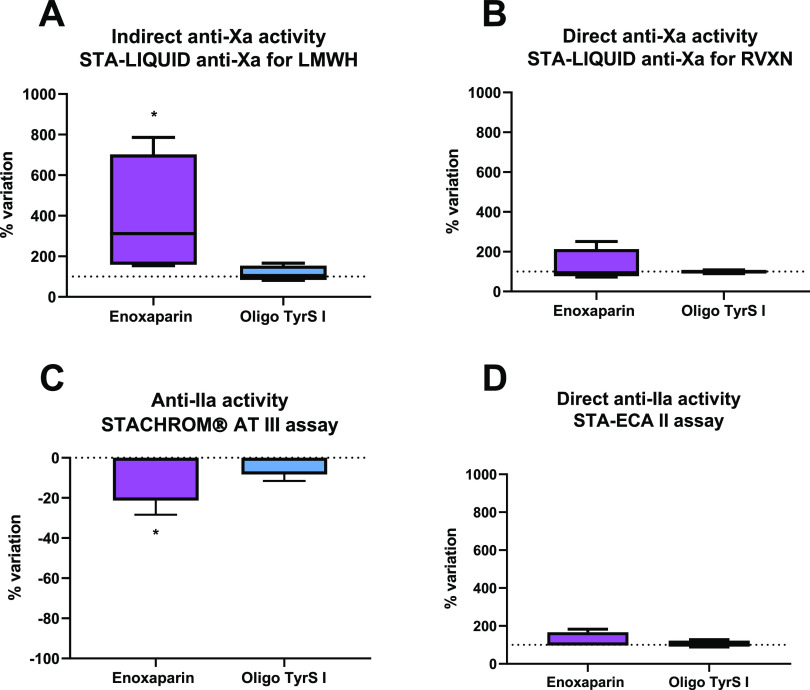
Effect of enoxaparin and OligoTyrS I on indirect (A) and direct (B) anti-Xa activities and indirect/direct (C) and direct (D) anti-IIa activities. Activities were measured in the plasma of CD1 mice treated with each compound using the (A) STA-LIQUID anti-Xa assay using the STA-Quality LMWH calibrator, (B) STA-LIQUID anti-Xa assay using the STA-Rivaroxaban Control, (C) STACHROM AT III assay, and the (D) STA-ECA II assay. Results are expressed as % of the value found in the plasma of nontreated mice. * represents a statistically significant effect (*p* < 0.05) compared with control mice (represented by the horizontal dotted line) (*n* = 4 each group). LMWH: low-molecular-weight heparin; RVXN: rivaroxaban.

To study the mechanism of action underlying the *in vivo* antithrombotic effect of OligoTyrS I, antithrombin-mediated inhibition of factors Xa and IIa was also investigated after *in vivo* administration. Therefore, OligoTyrS I was injected, using the same experimental conditions (dose and time of exposure), in another group of CD1 mice, but this time, instead of inducing thrombus formation, the mice blood was collected to study the putative coagulation enzymes affected. In contrast to purified enzymes, this approach has the advantage of studying the mechanism of action in a system where the enzymes/coagulation factors are at physiological concentrations and in a natural matrix (plasma). As shown in Figure 7, enoxaparin inhibited Xa activity indirectly, since it significantly affected the STA-LIQUID anti-Xa assay in comparison to nontreated control mice (horizontal dotted line), when the STA-Quality LMWH calibrator was used (Figure 7A). Also, the antithrombin activity was increased in the plasma of the enoxaparin-treated mice, since in the STACHROM AT III assay (Figure 7C), a significant reduction in the residual thrombin on the plasma of enoxaparin-treated mice was observed compared to that obtained with the plasma of nontreated control mice (horizontal dotted line). These assays are routinely used to monitor patients on enoxaparin therapy and patients with antithrombin deficiencies, respectively. However, OligoTyrS I, at the same experimental conditions (dose and time of exposure) that induced *in vivo* thrombus reduction, did not alter any of these assays, indicating that the thrombus reduction observed *in vivo* was not caused by antithrombin activation. We also evaluated a putative direct effect over factor Xa or IIa, using the STA-LIQUID anti-Xa assay with STA-Rivaroxaban Control (Figure 7B) and the STA-ECA II assay (Figure 7D). These assays are routinely used for monitoring patients on rivaroxaban and dabigatran therapies, respectively. We observed no alteration in comparison to nontreated control mice (horizontal dotted line) for OligoTyrS I as well as, as expected, to enoxaparin.

To further confirm that these negative results with OligoTyrS I were not due to pharmacokinetics issues of the *in vivo* assay, the control mice plasmas were pooled and spiked (1:10) with increasing concentrations of OligoTyrS I (final concentrations: 6.25, 12.5, 25, 50, and 100 mg/L). No alteration was observed with any of the concentration tested, while a concentration-dependent effect was still observed in APTT (data not shown).

These results point out that OligoTyrS I, combining both an interesting APTT anticoagulant activity and a lack of both anti-Xa and anti-IIa activities, had an effect on the intrinsic pathway rather than on the common pathway. For now, it was possible to disclose that antithrombins, FXa or FIIa, are not the targets for OligoTyrS I, but further assays would be needed to look for other targets that could be involved. In this context, it is interesting to report that Desai and colleagues^57^ identified the heparin-binding site of factor XIa (FXIa) as the target for a sulfated pentagalloylglucoside. Targeting proteases of the intrinsic pathway, especially FXIa, has been put forward as a powerful route to safer antithrombotics than those that inhibit factor Xa and thrombin.^59^ Therefore, the heparin-binding site for FXIa should be taken into account in future studies.

## Conclusions

The heterogeneous structure of heparins and their source in animal tissues explain how mandatory is to open access to structurally controlled non-natural sulfated species. Recently, the interest in the design and exploitation of highly sulfated bioactive polyphenols that could be a valuable alternative for application as anticoagulants has increased. In this context, the sulfated polymer of tyrosol, OligoTyrS I, was prepared from OligoTyr, one of the most representative phenolic constituents of extra virgin olive oil, and characterized in the present study. Sulfation allowed us to improve the water solubility, hence broadening the application fields of OligoTyr, previously described^50^ as an antioxidant material endowed with osteogenic activity. OligoTyrS I, with the alcoholic group sulfated and with the phenolic groups free, stands as a promising lead compound acting simultaneously as antioxidant and anticoagulant with proven antithrombotic effect *in vivo*. Thrombosis being a complex process involving multiple pathways, homogeneous sulfated phenolic polymers acting simultaneously as antioxidants and anticoagulants could be of value to prevent and treat this pathology.^60,61^ In addition, the lack of both anti-Xa and anti-IIa activities adding to the APTT anticoagulant activity showed that OligoTyrS I had an effect on proteases of the intrinsic pathway rather than on the common pathway, which have been pointed out as powerful targets to safer antithrombotics, able to reduce thrombotic complications, while leaving the hemostatic process largely intact.

In conclusion, the results reported in this study may stimulate further research aimed at assessing the potential of sulfated phenolic polymers as alternatives to heparins for biomedical purposes.

## Supporting Information Available

Elutographic profile of the reaction mixture of tyrosol; NMR spectra and ^1^H/^13^C resonances of TyrS; NMR spectra of OligoTyrS I and OligoTyrS II; MALDI-MS spectra of OligoTyrS I; UV–vis spectra; specific absorption coefficient of different batches of OligoTyrS I (PDF)

## Abbreviations Used

Tyr: tyrosol
TyrS: sulfated tyrosol
(SO_3_-TEA): sulfur trioxide triethylamine complex
EDTA: ethylenediaminetetraacetic acid
MALDI-MS: matrix-assisted laser desorption/ionization-mass spectrometry
DPPH: 2,2-diphenyl-1-picrylhydrazyl
FRAP: ferric reducing/antioxidant power
APTT: thromboplastin time
PT: prothrombin time
TT: thrombin time
TPTZ: 2,4,6-tri(2-pyridyl)-s-triazine
LMWH: low-molecular-weight heparin
HRP: peroxidase from horseradish
