# Supplementary material for: Sulfated Oligomers of Tyrosol: Toward a New Class of Bioinspired Nonsaccharidic Anticoagulants
Source: Biomacromolecules. 2021 Jan 12;22(2):399–409. doi: 10.1021/acs.biomac.0c01254 (PMC8023584; doi:10.1021/acs.biomac.0c01254)
Supplement: Supplementary file 1 — bm0c01254_si_001.pdf [file bm0c01254_si_001.pdf]

Sulfated oligomers of tyrosol: toward a new class of bioinspired non-saccharidic anticoagulants

Maria Laura Alfieri,[†] Lucia Panzella,[†] Bárbara Duarte,[⊥] Salomé Gonçalves-Monteiro,[§] Franklim Marques,[⊥] Manuela Morato,[§] Marta Correia-da-Silva,[‡] Luisella Verotta,[‖] and Alessandra Napolitano[†]*

[†]Department of Chemical Sciences, University of Naples Federico II, I-80126 Naples, Italy.

[⊥]UCIBIO/REQUIMTE and Clinical Analysis Unit, Department of Biological Sciences, Faculty of Pharmacy, University of Porto, 4050-313 Porto, Portugal.

[§]LAQV/REQUIMTE and Laboratory of Pharmacology, Department of Drug Sciences, Faculty of Pharmacy, University of Porto, 4050-313 Porto, Portugal.

[‡]CIIMAR and Laboratory of Organic and Pharmaceutical Chemistry, Department of Chemical Sciences, Faculty of Pharmacy, University of Porto, 4050-313 Porto, Portugal.

[‖]Department of Chemistry, University of Milan, 20133 Milano, Italy.

Table of contents

Figure S1. Elutographic profile of the reaction mixture of tyrosol. S2
Figure S2. ^{1}H NMR spectrum of TyrS in D_2O. S3
Figure S3. ^{13}C NMR spectrum of TyrS in D_2O. S4
Figure S4. ^{1}H,^{1}H COSY spectrum of TyrS in D_2O. S5
Figure S5. ^{1}H,^{13}C HSQC spectrum of TyrS in D_2O. S6
Figure S6. ^{1}H,^{13}C HMBC spectrum of TyrS in D_2O. S6
Figure S7. ^{1}H (black) and ^{13}C (red) NMR resonances of TyrS. S7
Figure S8. ^{1}H NMR spectrum of OligoTyrS I in D_2O. S8
Figure S9. ^{1}H NMR spectrum of OligoTyrS I after treatment with EDTA. S8
Figure S10. Segmental spectra of the OligoTyrS I MALDI-MS spectrum. S9
Figure S11. MALDI-MS of representative batches of OligoTyrS I. S10
Figure S12. UV-vis spectra and specific absorption coefficient of different batches of OligoTyrS I. S11
Figure S13. Proton spectra of two representative batches of OligoTyrS I. S11
Figure S14. ^{1}H NMR spectrum of OligoTyrS II in D_2O. S12
Figure S15. ^{1}H,^{1}H COSY spectrum of OligoTyrS II in D_2O. S12
Figure S16. ^{1}H,^{13}C HSQC spectrum of OligoTyrS II in D_2O. S13
Figure S17. ^{1}H,^{13}C HMBC spectrum of OligoTyrS II in D_2O. S13
Figure S18. ^{13}C NMR spectrum of OligoTyrS II in D_2O. S14

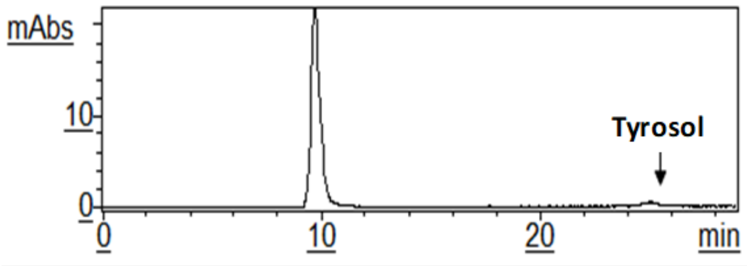

Figure S1. Elutographic profile of the reaction mixture of tyrosol in the presence of 5 molar equivalents of SO_3-TEA after 24 h. Detection wavelength 254 nm.

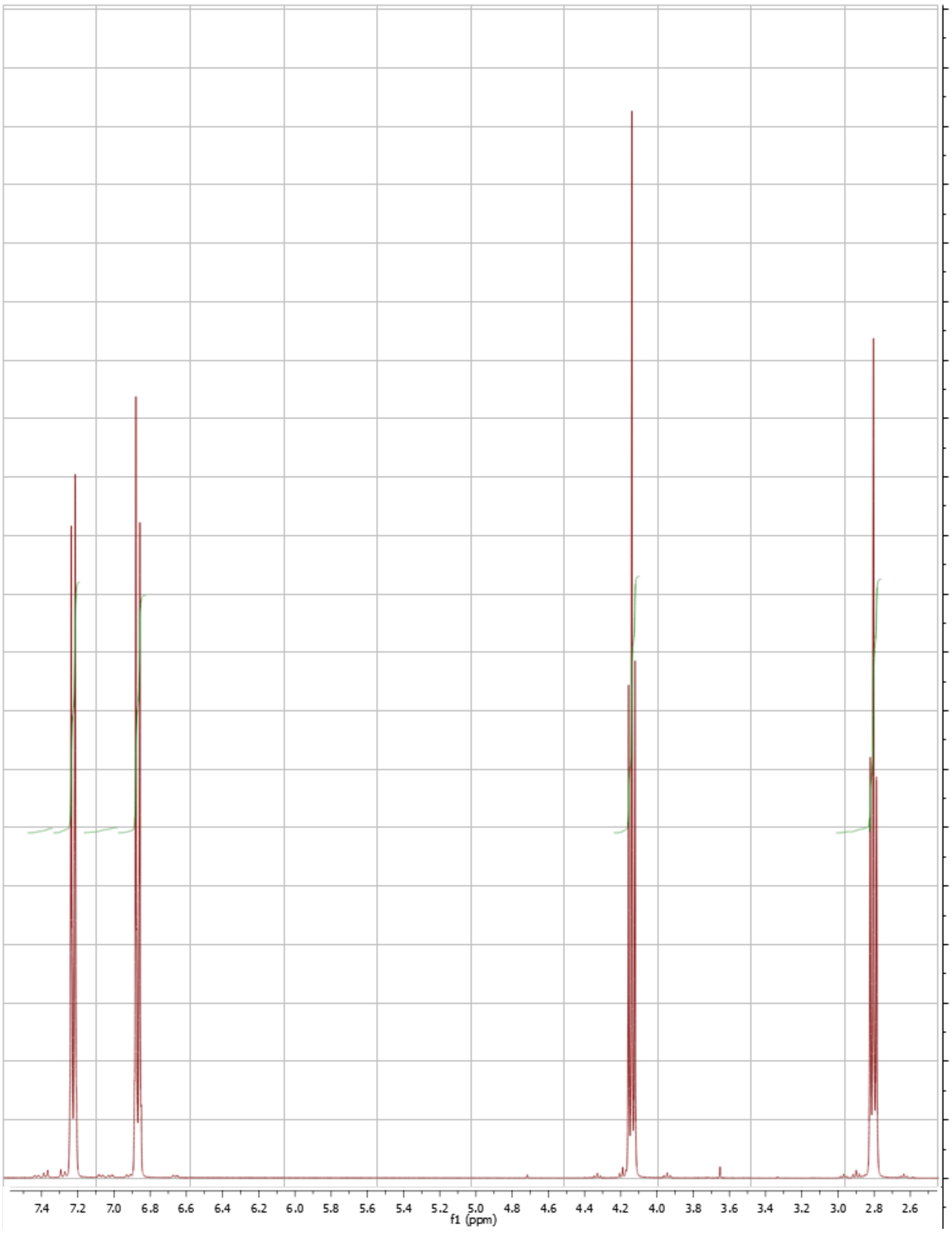

Figure S2. ^{1}H NMR spectrum of TyrS (400 MHz in D_2O).

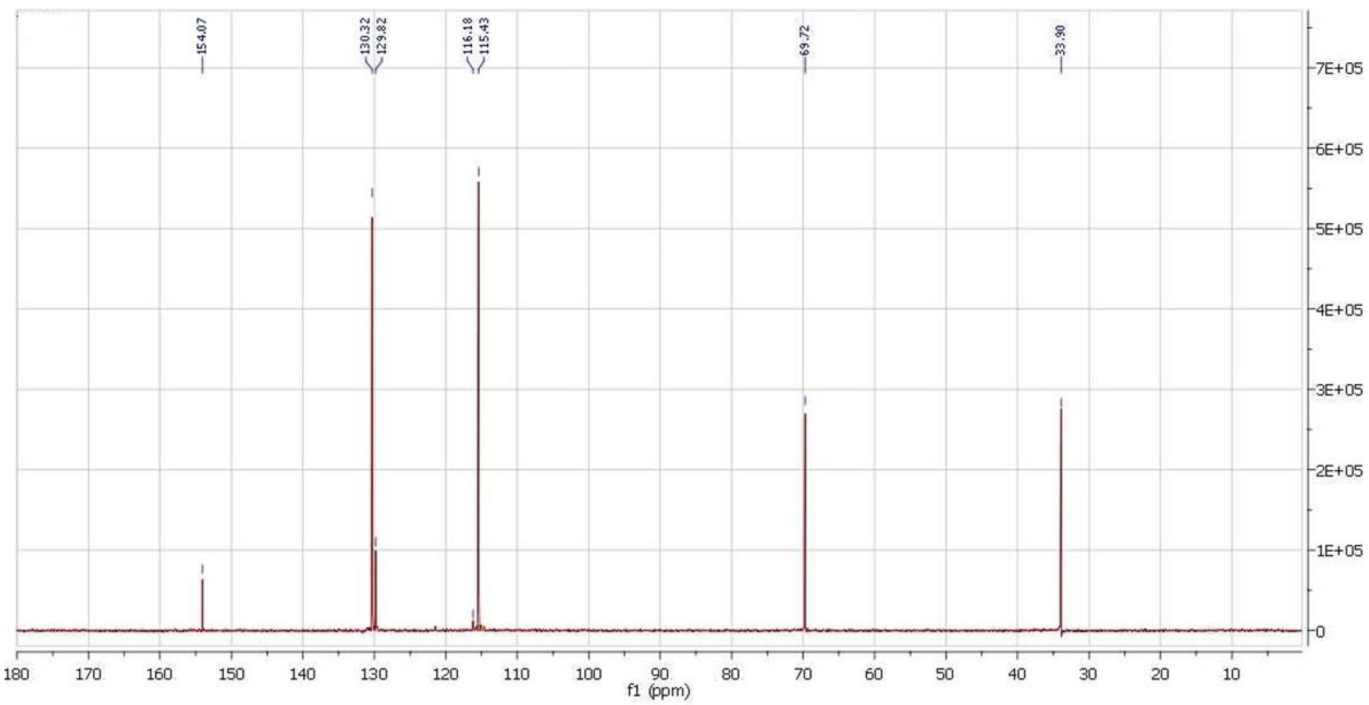

Figure S3. ^{13}C NMR spectrum of TyrS in D_2O.

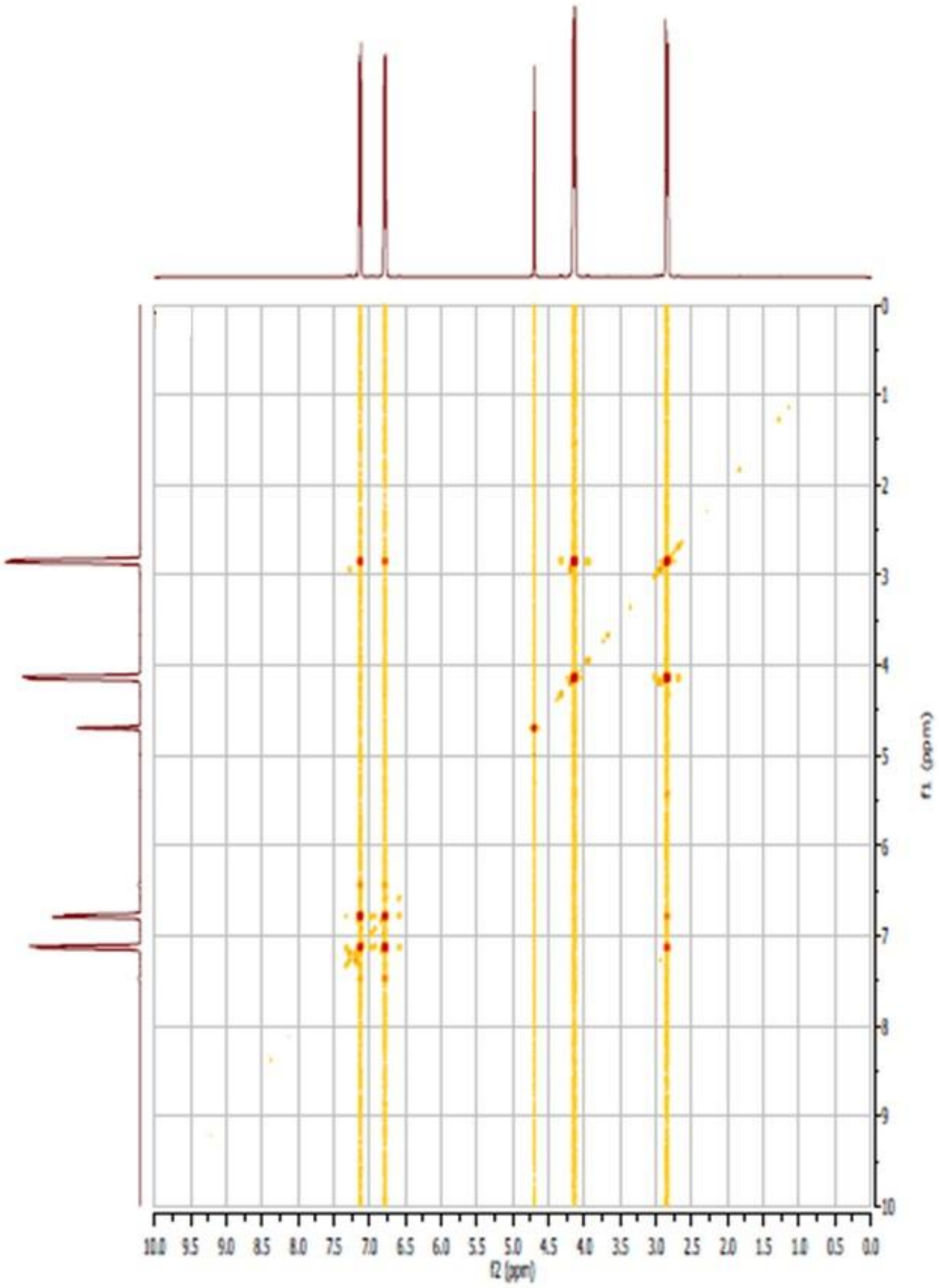

Figure S4. $^{1}H,^{1}H$ COSY spectrum of TyrS.

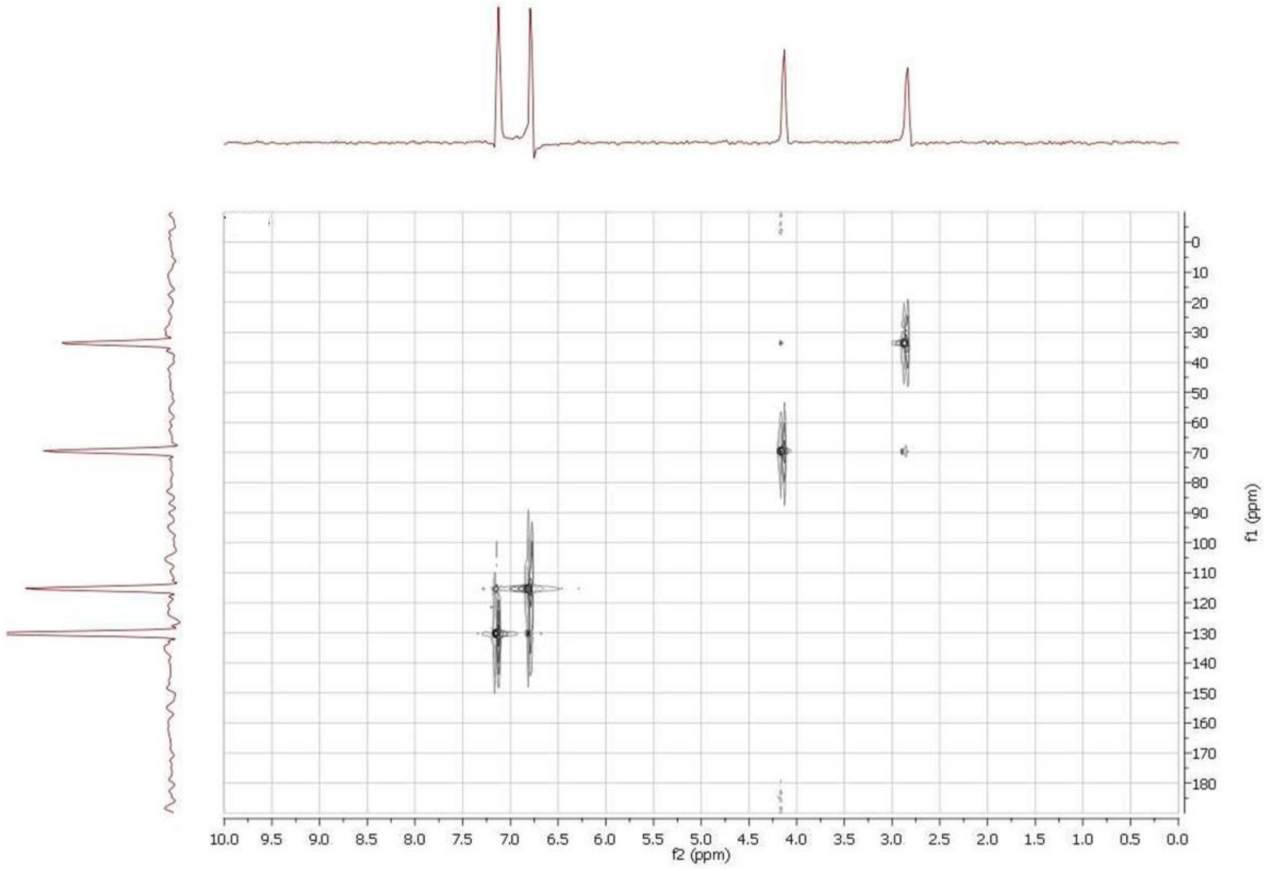

Figure S5. ^{1}H,^{13}C HSQC spectrum of TyrS.

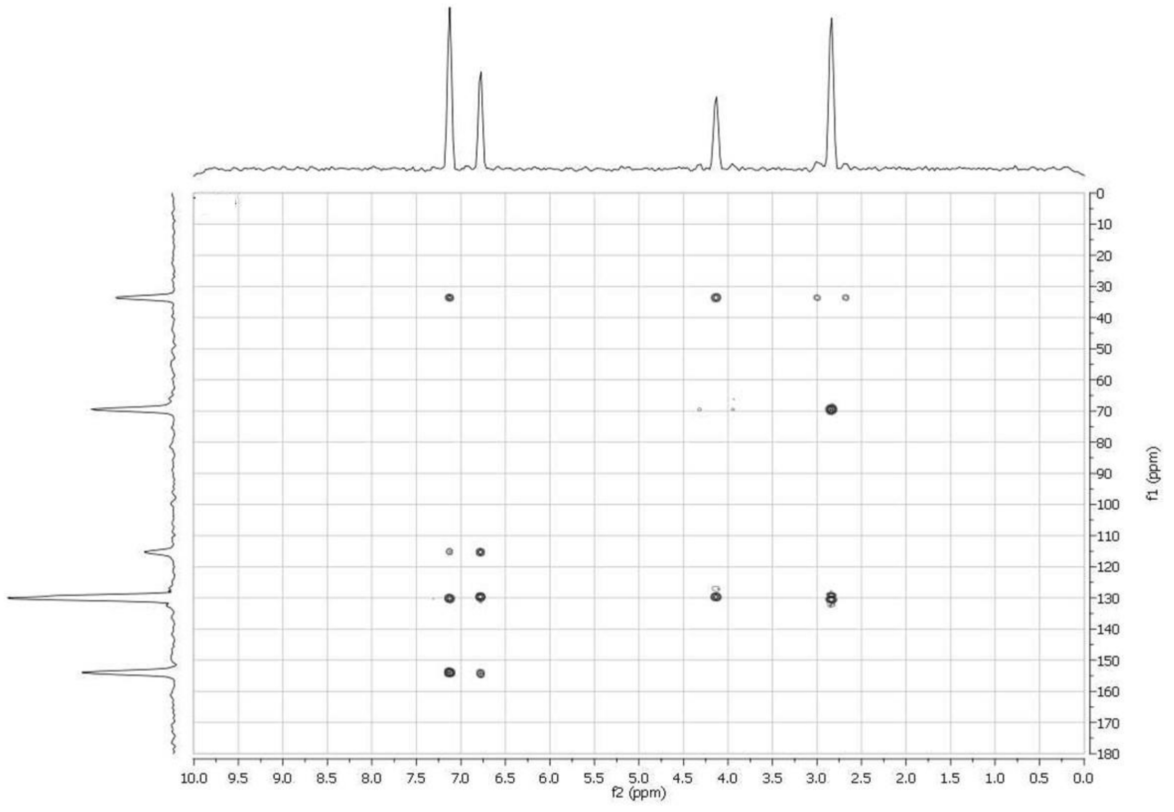

Figure S6. ^{1}H,^{13}C HMBC spectrum of TyrS.

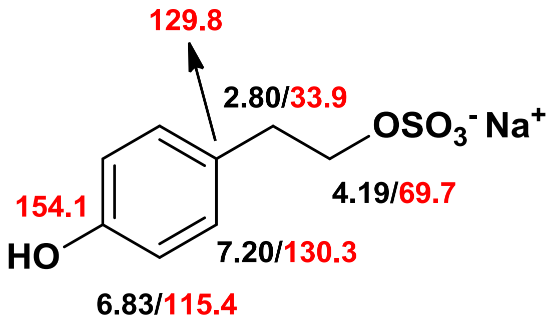

Figure S7. ^{1}H (black) and ^{13}C (red) NMR resonances of TyrS.

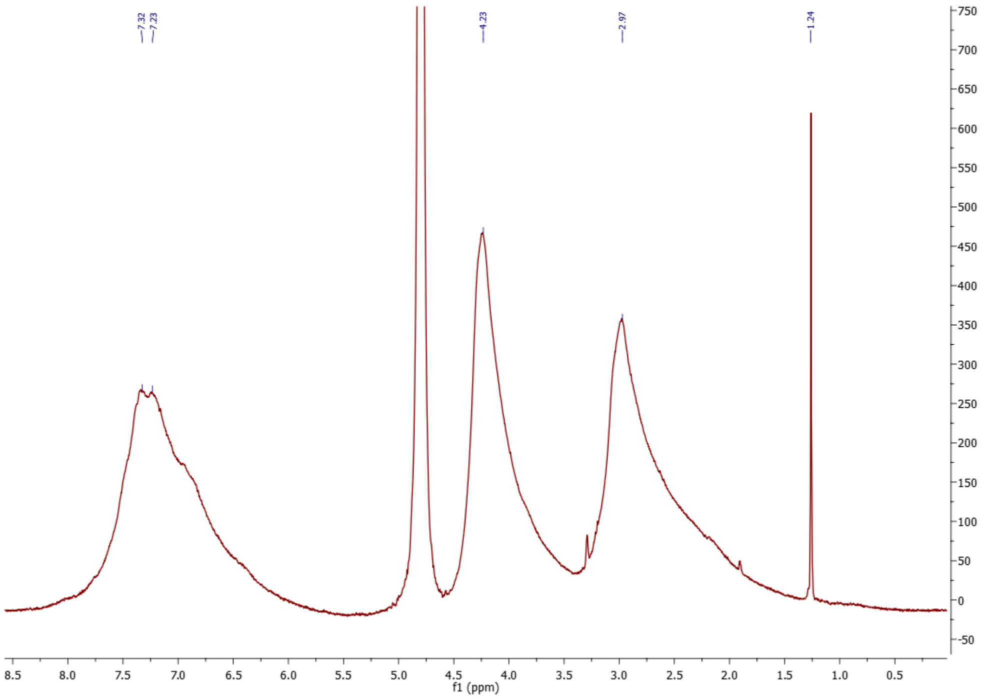

Figure S8. ^{1}H NMR spectrum of OligoTyrS I (400 MHz in D_2O).

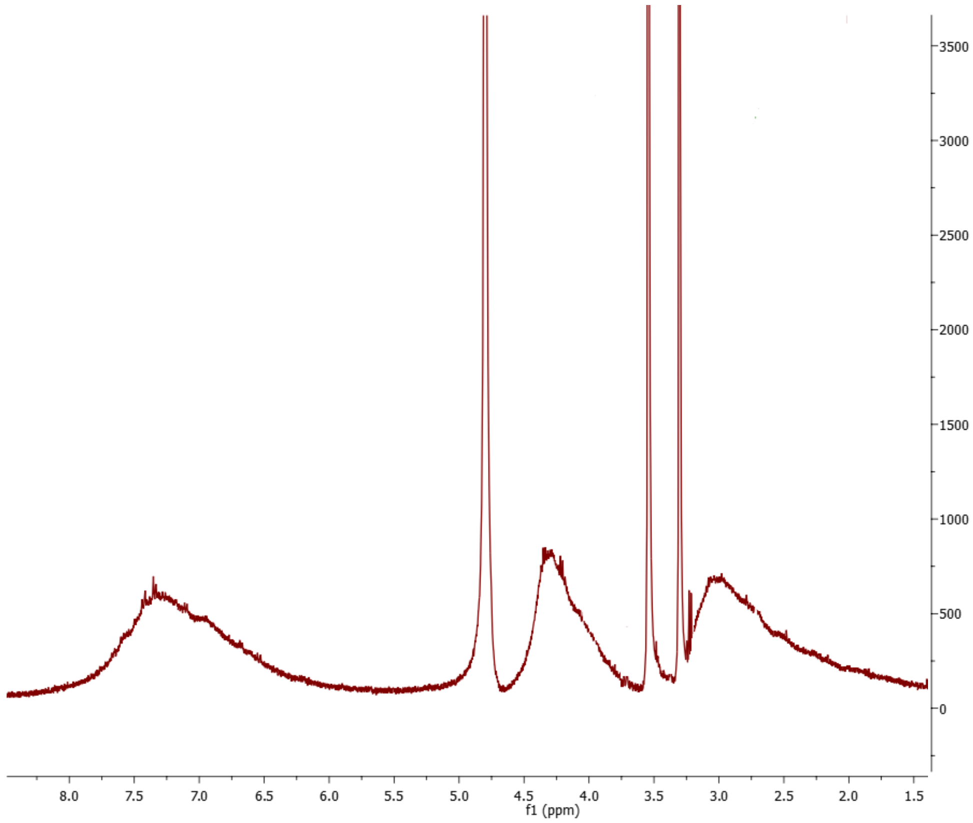

Figure S9. ^{1}H NMR spectrum of OligoTyrS I after treatment with ethylenediaminetetraacetic acid (EDTA).

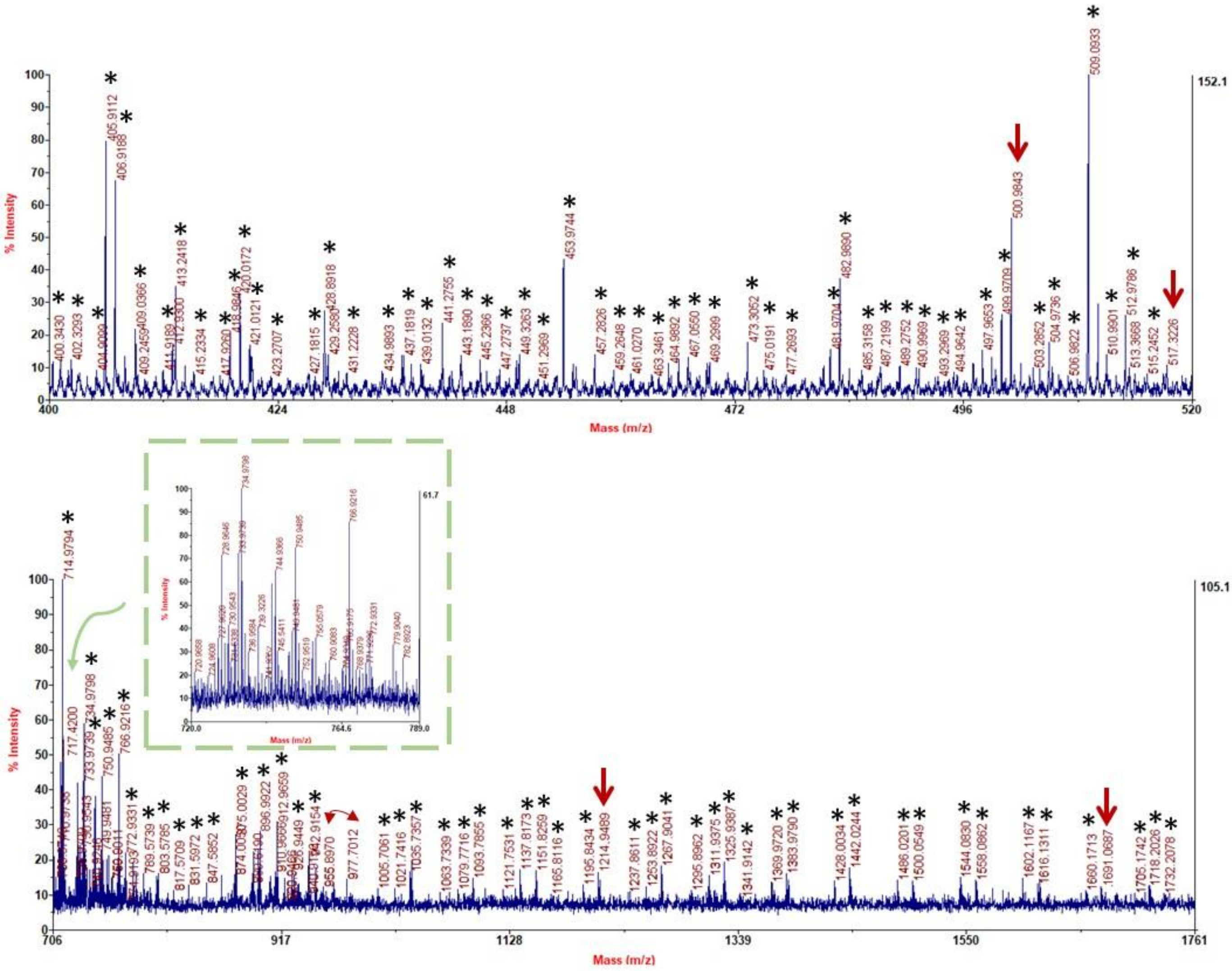

Figure S10. Segmental spectra of the OligoTyrS I MALDI-MS spectrum. Arrows indicate signals due to sulfated oligomers. Asterisks indicate signals due to matrix or impurities.

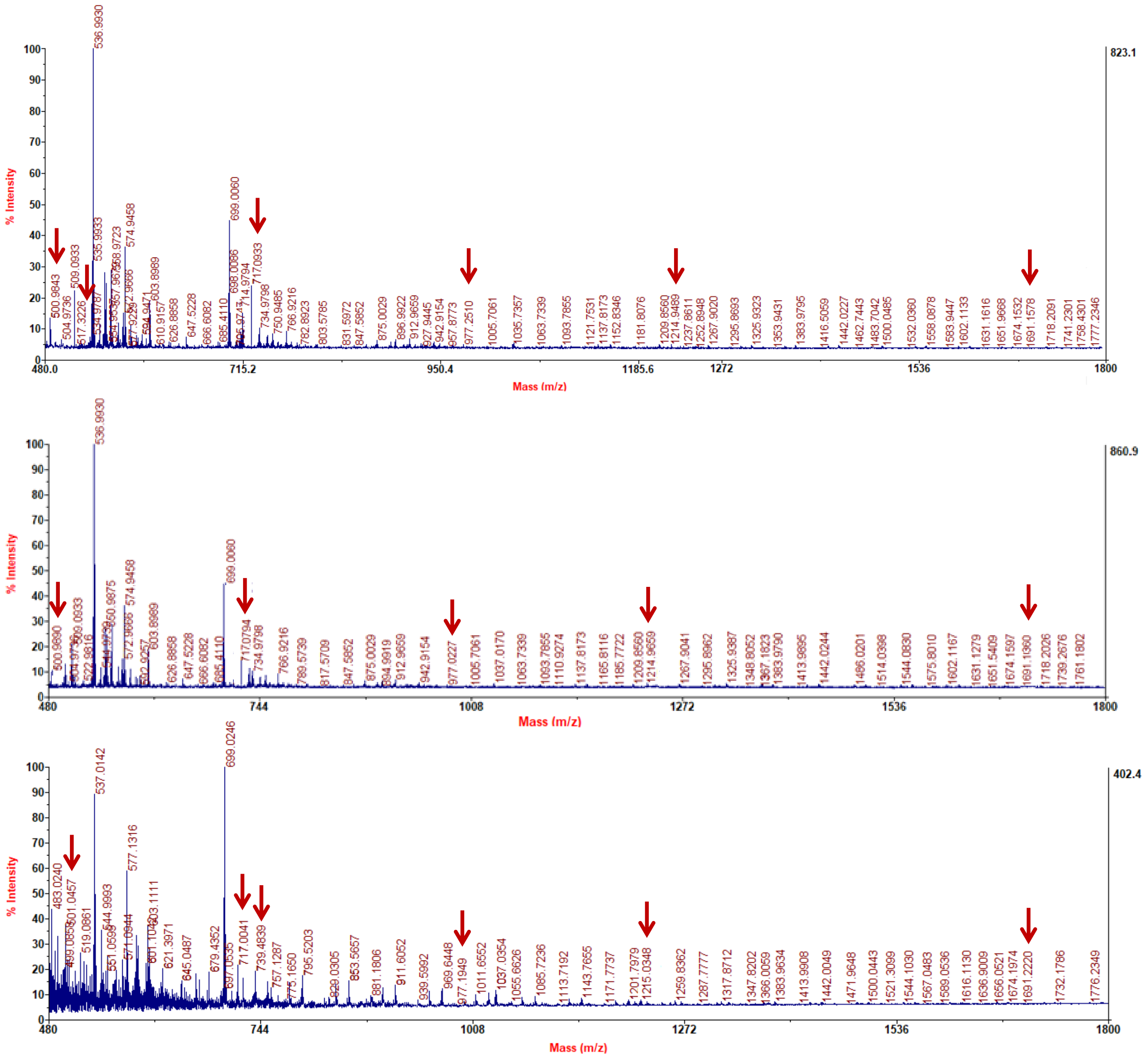

Figure S11. MALDI-MS of representative batches of OligoTyrS I. Arrows indicate signals due to sulfated oligomers.

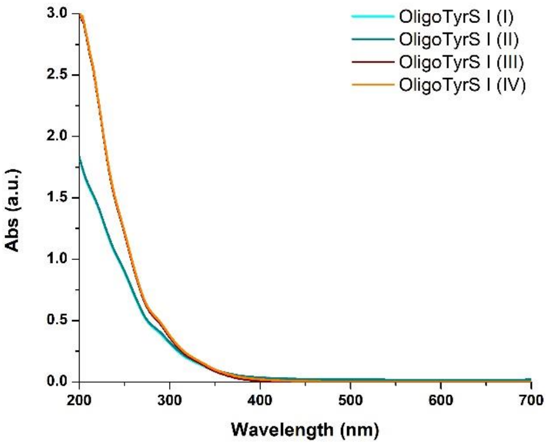

Batch #	ε (mg/mL)$^{-1}$ cm^{-1} (280 nm)
1	18.01
2	18.13
3	18.08
4	18.19

Figure S12. UV-vis spectra and specific absorption coefficient of different batches of OligoTyrS I.

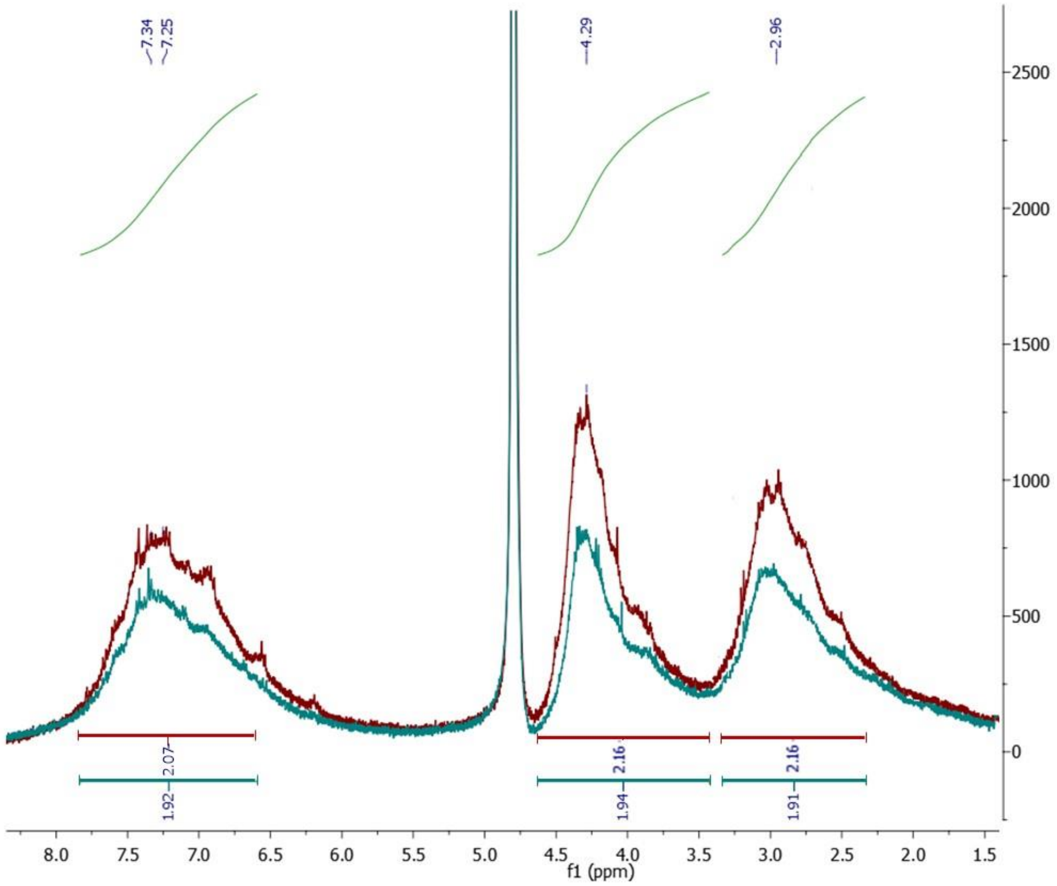

Figure S13. Proton spectra of two representative batches of OligoTyrS I. Shown are integration of peak areas for each spectrum.

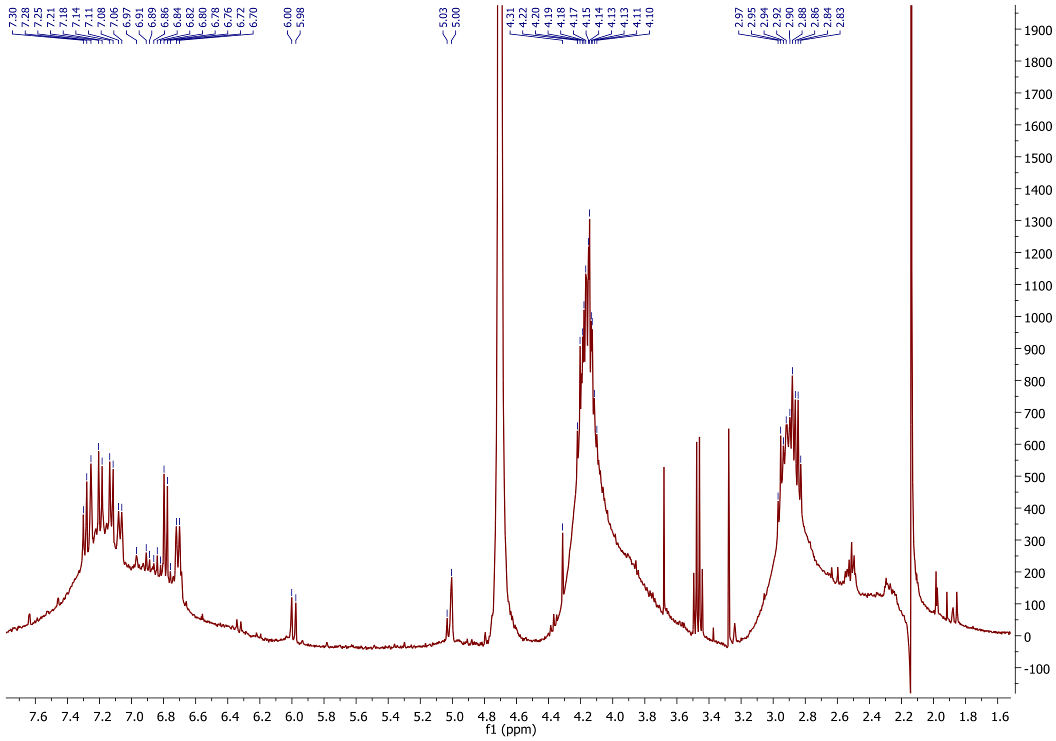

Figure S14. ^{1}H NMR spectrum of OligoTyrS II (400 MHz in D_2O).

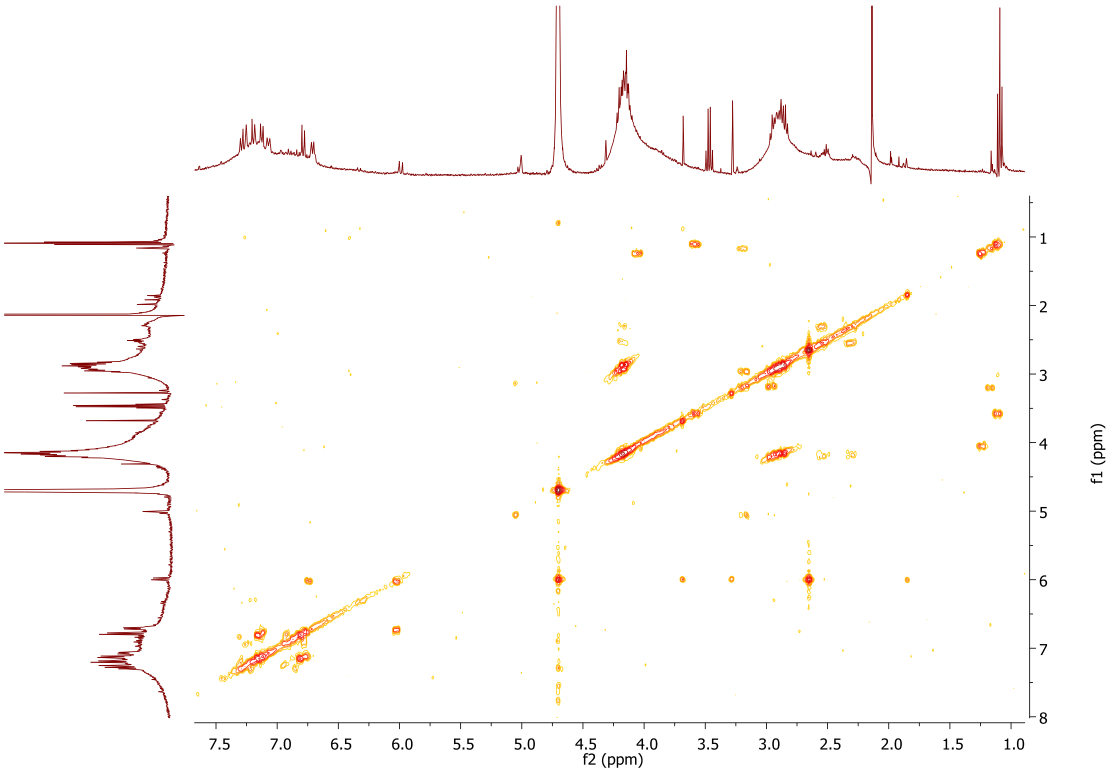

Figure S15. $^{1}H,^{1}H$ COSY spectrum of OligoTyrS II.

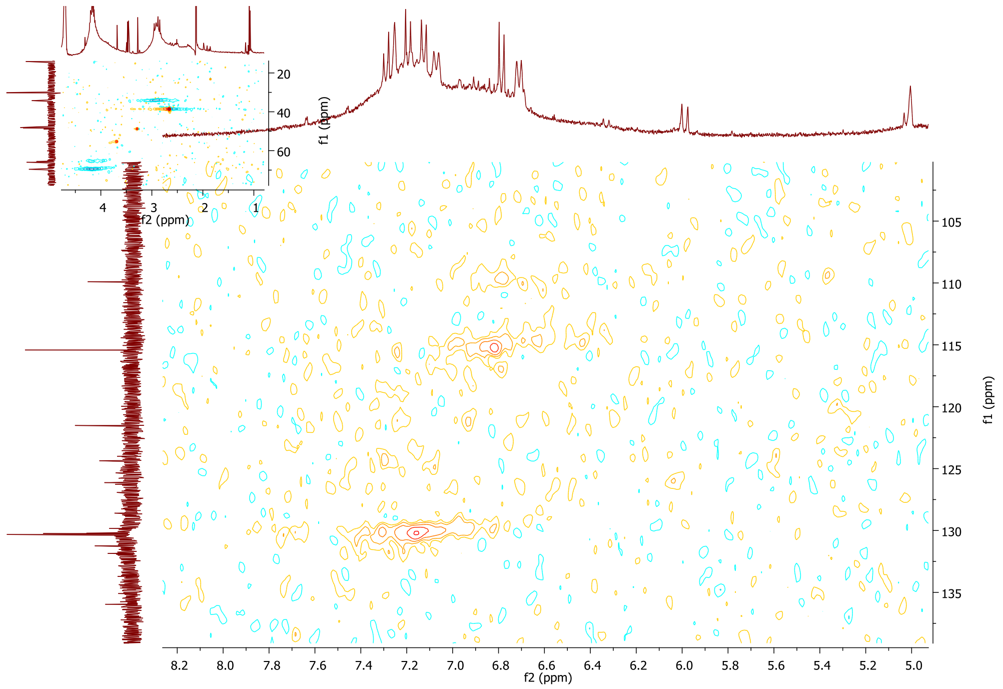

Figure S16. ^{1}H,^{13}C HSQC spectrum of OligoTyrS II.

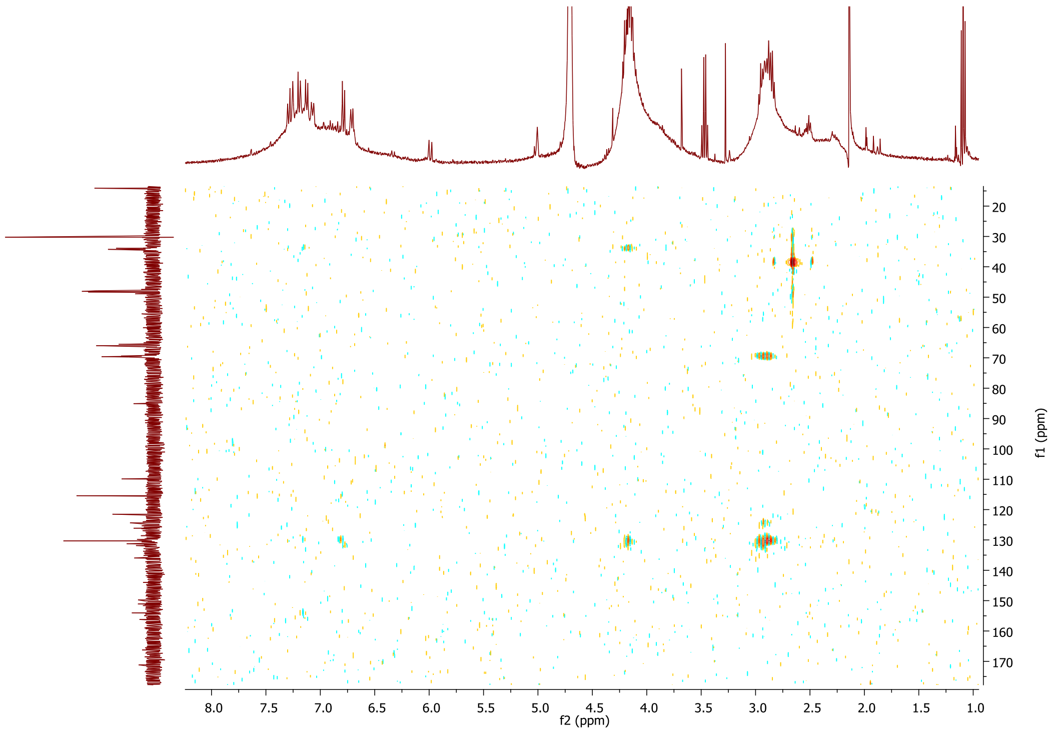

Figure S17. ^{1}H,^{13}C HMBC spectrum of OligoTyrS II.

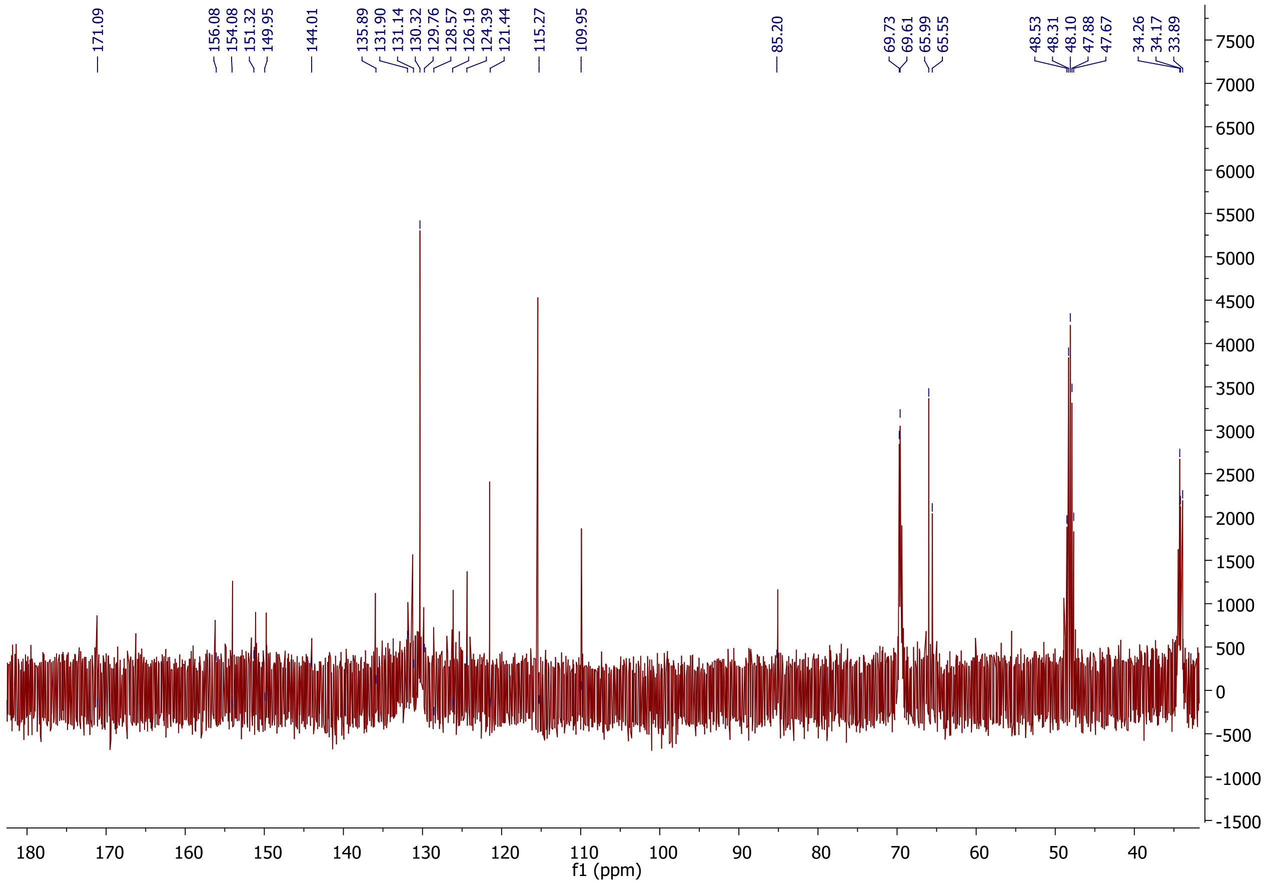

Figure S18. ^{13}C NMR spectrum of OligoTyrS II in D_2O.
